# Dissemination of *Ras*^*V12*^-transformed cells requires the mechanosensitive channel Piezo

**DOI:** 10.1038/s41467-020-17341-y

**Published:** 2020-07-16

**Authors:** Jiae Lee, Alejandra J. H. Cabrera, Cecilia M. T. Nguyen, Young V. Kwon

**Affiliations:** Department of Biochemistry, University of Washington, Seattle, WA 98195 USA

**Keywords:** Cell invasion, Cell signalling, Disease model

## Abstract

Dissemination of transformed cells is a key process in metastasis. Despite its importance, how transformed cells disseminate from an intact tissue and enter the circulation is poorly understood. Here, we use a fully developed tissue, *Drosophila* midgut, and describe the morphologically distinct steps and the cellular events occurring over the course of *Ras*^*V12*^-transformed cell dissemination. Notably, *Ras*^*V12*^-transformed cells formed the Actin- and Cortactin-rich invasive protrusions that were important for breaching the extracellular matrix (ECM) and visceral muscle. Furthermore, we uncovered the essential roles of the mechanosensory channel Piezo in orchestrating dissemination of *Ras*^*V12*^-transformed cells. Collectively, our study establishes an in vivo model for studying how transformed cells migrate out from a complex tissue and provides unique insights into the roles of Piezo in invasive cell behavior.

## Abstract (web summary)

Drosophila tumours can be utilised to study the mechanisms of cell dissemination. Here, the authors use *Drosophila* midgut to examine the course of *Ras*^*V12*^-transformed cell dissemination from midgut into circulation, which requires the actions of invasive protrusions and the mechanosensitive channel Piezo.

## Introduction

A hallmark of malignancy, metastasis is the major contributor of mortality in cancer patients^1,2^. To metastasize, cancer cells undergo a series of processes, including dissemination from the original tumor site, circulation in blood or lymph, extravasation, and then colonization at a secondary site^2,3^. Given that metastasis is initiated by dissemination from the original tumor site into the circulation, targeting this initial step could be an ideal strategy for intervening metastasis. Most of our knowledge of the molecular mechanisms of cell dissemination has been acquired from studies utilizing cancer cells in culture. Nevertheless, recapitulating the intricacy of the native microenvironment using in vitro culture systems remains difficult at best. Therefore, a simple in vivo system allowing us to observe the dissemination process in a native context will be useful to achieve a better understanding on the mechanisms that transformed cells utilize for dissemination.

Simple model organisms have provided tools to investigate the mechanisms underlying cell migration and invasion in native contexts^4^. For instance, migration of the border cells during *Drosophila* oocyte development has illustrated how cells can migrate collectively^4,5^. In addition, invasion of *Caenorhabditis elegans* (*C. elegans*) anchor cells into the vulval epithelia has provided a powerful tool to study invasive cell behavior in an in vivo microenvironment^6,7^. In *Drosophila*, genetic manipulation of epithelial tissues can induce hyperplasia or tissue overgrowth—a so called ‘tumor’ in *Drosophila*^8–11^. Notably, these *Drosophila* tumors also provide a tool to study metastatic behavior^12,13^. In particular, a recent study demonstrated that adult hindgut epithelial cells expressing mutant *Ras* (*Ras*^*V12*^) could disseminate from the hindgut and metastasize to distant tissues^14,15^. Interestingly, the same study elucidated that sustained intestinal infection with pathogenic bacteria could enhance dissemination of *Ras*^*V12*^-expressing hindgut cells via activation of Imd innate immune signaling^14^. Although these tumor models have allowed the identification of several genetic and environmental factors underlying metastatic phenotypes^12–14,16,17^, it still remains obscure how these transformed cells disseminate from the primary tumor site into the hemocoel to initiate metastasis.

Here, we show that expression of *Ras*^*V12*^ in intestinal stem cells (ISCs) and enteroblasts (EBs) in the adult *Drosophila* midgut causes them to disseminate from the posterior midgut and transmigrate into the circulation. Our cellular and molecular characterization reveals how some of the molecular mechanisms underlying the migratory and invasive phenotypes of cancer cells are assembled in vivo to form a mode of cell dissemination. Observing the cell dissemination process in a native context allows us to describe actin- and cortactin-rich invasive protrusions that are associated with degradation of the ECM and the visceral muscle (VM) layer in *Drosophila* and discover the mechanosensitive channel Piezo as a key player of cell dissemination in vivo.

## Results

### *Ras*^*V12*^ cells basally disseminate from the posterior midgut

**Fig. 1:**
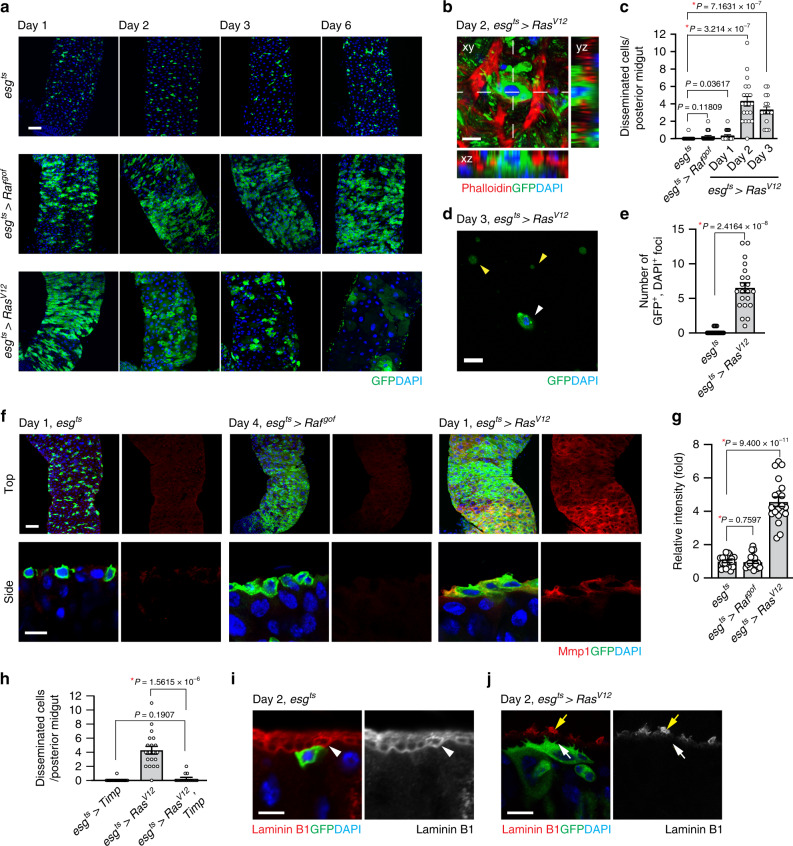
*Ras*^*V12*^ cells basally disseminate from the posterior midgut. **a** Images of the posterior midgut. Transgenes were induced with *esg*^*ts*^ by incubating at 29 °C for indicated durations. The cells manipulated by *esg*^*ts*^ are marked and stained with GFP (green), and nuclei are stained with DAPI (blue). Scale bar, 50 µm. **b** Representative image of disseminated cell. Top view (xy) and orthogonal views (yz and xz) are shown. Phalloidin (red) visualizes VM. Scale bar, 10 µm. **c** Quantification of disseminated cells detected on the surface of posterior midgut. *N* = 25 (*esg*^*ts*^), *N* = 20 (*esg*^*ts*^ > *Raf*^*gof*^), *N* = 21 (day 1, *esg*^*ts*^ > *Ras*^*V12*^), *N* = 20 (day 2, *esg*^*ts*^ > *Ras*^*V12*^), *N* = 16 (day 3, *esg*^*ts*^ > *Ras*^*V12*^) biological replicates. **d** Representative image of GFP^+^ and DAPI^+^ cell (white arrowhead) detected in hemolymph. *Ras*^*V12*^ was expressed with *esg*^*ts*^ for 3 days before hemolymph collection. GFP^+^ and DAPI^–^ particles (yellow arrowheads) were also detected. Scale bar, 10 µm. **e** Quantification of circulating GFP^+^ and DAPI^+^ cells. *N* = 3 independent experiments for each genotype. **f** Mmp1 immunostaining (red) in posterior midguts. Lower panels show side view of the cells. Scale bars, 50 µm (top) and 10 µm (bottom). **g** Quantification of Mmp1 levels. *N* = 20 data points collected from seven biological replicates for each genotype. **h** Quantification of disseminated cells detected on the surface of posterior midgut. *N* = 16 (*esg*^*ts*^ > *Timp*), *N* = 20 (*esg*^*ts*^ > *Ras*^*V12*^), *N* = 14 (*esg*^*ts*^ > *Ras*^*V12*^*, Timp*) biological replicates. **I**–**j** Laminin B1 staining of midguts. *esg*^*ts*^ and *esg*^*ts*^ > *Ras*^*V12*^ midguts were stained with anti-laminin B1 antibody. White arrowhead in the *esg*^*ts*^ image points to the inner laminin layer adjacent to the epithelium, white arrow in the *esg*^*ts*^ > *Ras*^*V12*^ images points the boundary of the epithelium where laminin is degraded, and yellow arrow in the *esg*^*ts*^ > *Ras*^*V12*^ images points to a patchy laminin signal outside VM. Scale bar, 10 µm. In the side views, the basal side of epithelia is positioned upward. In (**c**), (**e**), (**g**), and (**h**), mean ± SEMs are shown with individual data points. Data were analyzed by two-tailed unpaired Student’s *t*-test. Asterisks indicate statistical significance (*P < 0.01) and *P* values are indicated in graph.

*Ras* genes encode small GTPases that are frequently mutated in multiple types of cancers^18^. Oncogenic Ras isoforms affect multiple aspects of cancers, including the metastatic transformation of breast cancers^19–21^. In *Drosophila*, ectopic expression of *Ras*^*V12*^ in developing disks increases cell division; however, it is not sufficient to induce malignant transformation. Disruption of polarity in addition to *Ras*^*V12*^ is required to induce malignant disc tumors with metastatic properties^12^. Similarly, ectopic expression of *Ras*^*V12*^ in midgut ISCs and EBs using a clonal strategy was not sufficient to induce tumors. Instead, *Ras*^*V12*^-expressing cells gradually disappeared from midguts^22,23^. When we expressed *Ras*^*V12*^ in adult midgut ISCs and EBs using the conditional GAL4 driver *esg*^*ts*^ (*escargot-GAL4*, *tubulin-GAL80*^*ts*^, *UAS-GFP*/+; see Methods), *Ras*^*V12*^ cells propagated initially and then, progressively disappeared from the midgut. At day 6 of *Ras*^*V12*^ expression, most of the *Ras*^*V12*^ cells had been eliminated from the midgut (Fig. 1a). In contrast, cells expressing a gain-of-function *Raf* allele (*Raf*^*gof*^) were retained in the midgut (Fig. 1a), indicating that general alteration in cell proliferation or cell crowding is not the cause of the phenotype.

Previously, it has been reported that the hindgut epithelial cells expressing *Ras*^*V12*^ could disseminate and metastasize to distant tissues^14^. Similarly, we noticed that a significant number of GFP-labeled *Ras*^*V12*^ cells were detected outside of the VM at day 2 of *Ras*^*V12*^ expression (Fig. 1b, c). Moreover, we detected GFP-labeled cells in hemolymph prepared from flies expressing *Ras*^*V12*^ with *esg*^*ts*^, but not from control flies (Fig. 1d, e). To be detected outside the VM or in hemolymph, these cells must have passed through the basement membrane (BM), which resides outside of the midgut epithelium.

Previous studies have shown that metastatic transformation in *Drosophila* can upregulate Matrix-metalloprotease 1 (Mmp1)^14,16,24^, which plays a crucial role in the degradation of the extracellular matrix (ECM). Similarly, we found that Mmp1 levels were increased by expression of *Ras*^*V12*^ but not *Raf*^*gof*^ (Fig. 1f, g). In addition, expression of *Ras*^*V12*^ with *esg*^*ts*^ also caused a cell non-autonomous increase in Mmp1 signals in surrounding cells. Although expression of MMPs was thought to be critical for invasive cell behavior, anchor cells in *C. elegans* could physically breach the basement membrane even in the absence of MMPs by employing an extensive F-actin-rich protrusion^25^. We found that expression of Tissue inhibitor of metalloproteases (Timp) with *esg*^*ts*^ completely suppressed dissemination of *Ras*^*V12*^ cells (Fig. 1h and Supplementary Fig. 1a), suggesting the importance of MMPs in dissemination of *Ras*^*V12*^ cells.

Adult midgut is surrounded by thick ECM, which can be visualized by staining laminin: one continuous laminin layer is detected at the basal side of the epithelium (Fig. 1i and Supplementary Fig. 1c, white arrowhead) and another prominent laminin layer is located at the outside of the VM (Supplementary Fig. 1c, yellow arrowhead). This complexity in the midgut ECM structure might account for the requirement of MMPs for dissemination of *Ras*^*V12*^ cells. In accordance with Mmp1 elevation, we detected deterioration of laminin structure. At day 1 of *Ras*^*V12*^ expression, we frequently observed a partial degradation of the ECM, which is manifested by loss of the laminin layer adjacent to the intestinal epithelium (Supplementary Fig. 1c, white arrow). At day 2 of *Ras*^*V12*^ expression, more extensive degradation affecting the entire ECM structure was observed. The inner laminin layer juxtaposed with the intestinal epithelium was almost completely absent (Fig. 1j, white arrow). Moreover, the laminin layer outside visceral muscle was also significantly compromised, leading to patchy pattern of laminin signals outside of the visceral muscle at day 2 of *Ras*^*V12*^ expression (Fig. 1j, yellow arrow and Supplementary Fig. 1d). Of importance, laminin signals were almost invisible at the basal side of disseminated *Ras*^*V12*^ cells (Supplementary Fig. 1c, white arrow). This global degradation of the laminin layer could be explained by the fact that *Ras*^*V12*^ cells covered the entire basal side of the midgut epithelium at day 1 of *Ras*^*V12*^ expression (Supplementary Fig. 2b). Therefore, a tissue-wide upregulation of Mmp1 signals was detected at day 1 of *Ras*^*V12*^ expression (Fig. 1f). In addition, we speculate that cell non-autonomous MMP expression may contribute to the profound ECM degradation phenotype although the molecular mechanism underlying this cell non-autonomous Mmp1 expression requires further investigations.

We noticed that *Ras*^*V12*^ cells also frequently delaminated toward the midgut lumen after 2 days of *Ras*^*V12*^ expression (Supplementary Fig. 2b’). Altogether, our observations suggest that *Ras*^*V12*^ cells migrate out from the posterior midgut via at least two different processes: dissemination into the hemocoel and delamination into lumen. Of note, expression of anti-apoptotic protein p35 did not impair the dissemination of *Ras*^*V12*^ cells (Supplementary Fig. 2c), suggesting that cell death was not responsible for the phenotype.

### Disseminating *Ras*^*V12*^ cells undergo morphological remodeling

**Fig. 2:**
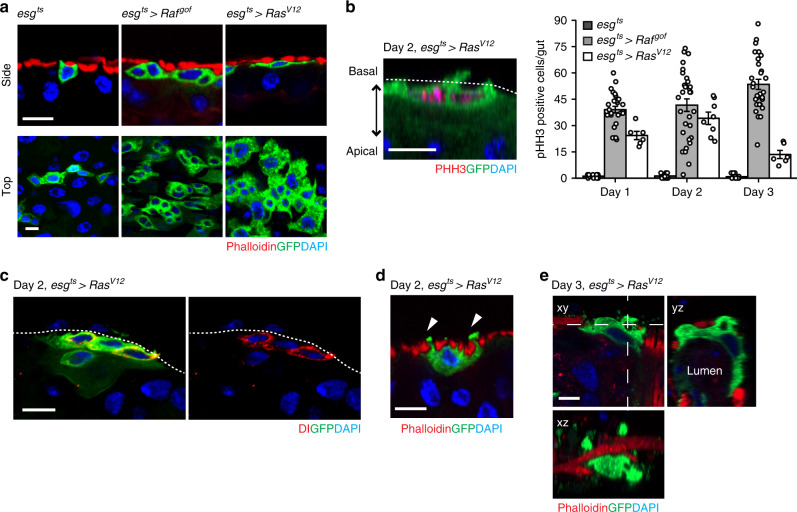
Disseminating *Ras*^*V12*^-transformed cells undergo extensive remodeling of cell morphology. **a** Representative cell morphologies from side and top view. Transgenes were induced for 1 day. Phalloidin signal visualizes VM (red). Scale bars, 10 µm. **b** Representative image of pHH3 (red) staining of *Ras*^*V12*^ cells and quantification of phospho-histone H3 (pHH3) cells per midgut after 3 days of expression. Prior to staining, *Ras*^*V12*^ was expressed for 2 days. Dotted line indicates the epithelial boundary. Scale bars, 10 µm. For quantification, day 1: *N* = 16 (*esg*^*ts*^), *N* = 27 (*esg*^*ts*^ > *Raf*^*gof*^), *N* = 6 (*esg*^*ts*^ > *Ras*^*V12*^); day 2: *N* = 14 (*esg*^*ts*^), *N* = 31 (*esg*^*ts*^ > *Raf*^*go*f^), *N* = 8 (*esg*^*ts*^ > *Ras*^*V12*^); day 3: *N* = 16 (*esg*^*ts*^), *N* = 29 (*esg*^*ts*^ > *Raf*^*gof*^), *N* = 6 (*esg*^*ts*^ > *Ras*^*V12*^) biological replicates. Data are mean ± SEMs. **c** Delta (Dl) antibody staining. The Notch receptor ligand Dl—a marker of intestinal stem cells—was detected in *Ras*^*V12*^ cells. Dotted line indicates the epithelial boundary. *Ras*^*V12*^ cells are marked with GFP (green), and nuclei are stained with DAPI (blue). Scale bar, 10 µm. **d** Side view of *Ras*^*V12*^ cell. *Ras*^*V12*^ was induced for 2 days with *esg*^*ts*^. Arrowheads point to protrusions formed across VM. Scale bar, 10 µm. **e** Side (xy) and orthogonal (yz and xz) views of *Ras*^*V12*^ cell. *Ras*^*V12*^ was expressed for 3 days. Scale bar, 10 µm.

Since the mechanism by which cells disseminate from a complex tissue is poorly described, we further investigated the phenomenon by scrutinizing the morphology of *Ras*^*V12*^ cells. We found that *Ras*^*V12*^ cells basally moved out from midgut epithelia at day 1 of *Ras*^*V12*^ expression (Fig. 2a). They resided in-between the epithelium and the VM layer and were significantly spread out (Fig. 2a). Interestingly, a significant portion of *Ras*^*V12*^ cells appeared to be bigger than normal ISCs or EBs (Supplementary Fig. 3a, b), an indication of possible misdifferentiation^26^. Nevertheless, a large portion of *Ras*^*V12*^ cells residing outside the epithelium could still divide (Fig. 2b) and express the Notch receptor ligand, Delta (Dl) (Fig. 2c and Supplementary Fig. 3c), which are the characteristics of active ISCs. At days 2 and 3 of *Ras*^*V12*^ expression, *Ras*^*V12*^ cells produced large protrusions across the VM while these large protrusions were not detected in control or *Raf*^*gof*^ cells (Fig. 2d). Furthermore, we were able to observe cells adopting various morphologies that appeared to be traversing the muscle layer (Fig. 2e).

### Disseminating *Ras*^*V12*^ cells assemble invasive protrusions

**Fig. 3:**
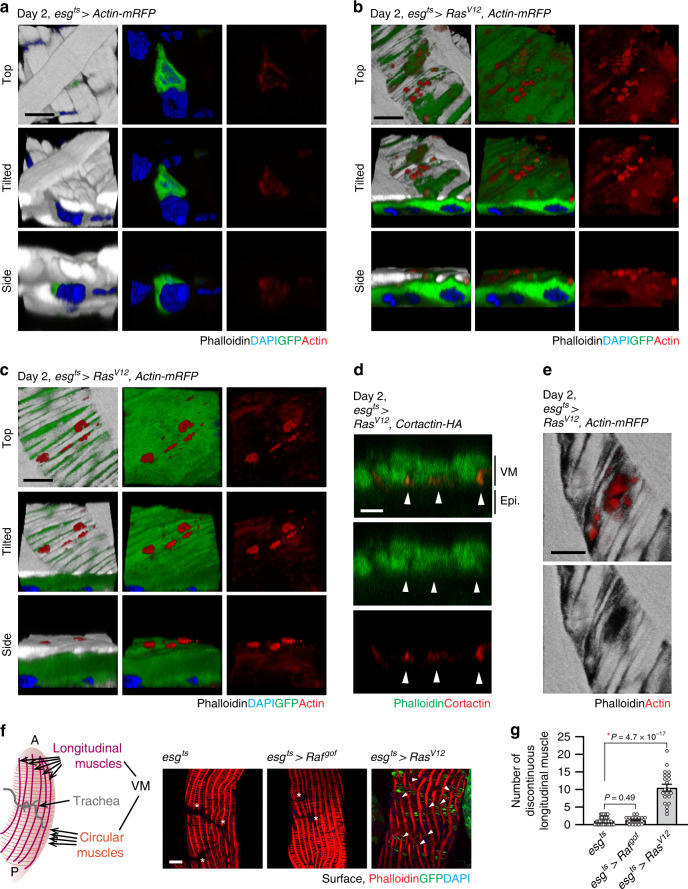
Basal actin-rich protrusions disrupt visceral muscle integrity. **a**–**c** 3D reconstructions of confocal images angled at 0, 45, and 90 degrees for top, tilted, and side views, respectively. Representative images of control cells (**a**), and *Ras*^*V12*^ cells (**b**, **c**) are shown. Dissected guts are imaged for actin-mRFP (red), phalloidin (gray), and DAPI (blue). Scale bars, 5 µm. **d** Orthogonal view of cortactin-rich protrusions and VM layer. Arrowheads indicate cortactin-rich protrusions (red) co-stained with phalloidin (gray). Scale bar, 5 µm. **e** Surface view of a rupture in the VM layer induced by a cluster of actin-rich protrusions (red). Scale bar, 5 µm. *N* = 12 (*esg*^*ts*^ > *Actin-mRFP*) for (**a**) and *N* = 28 (*esg*^*ts*^ > *Ras*^*V12*^*, Actin-mRFP*) biological replicates for (**b**), (**c**), (**d**), and (**e**). **f** Surface views of the posterior midgut. Schematic illustration describes the architecture of the *Drosophila* VM. VM (red) is visualized with phalloidin. Arrowheads show discontinued longitudinal muscles. The region void of phalloidin signals (asterisks) is where the trachea is present. Longitudinal and circular muscles behind trachea are not captured because they are at different focal planes. **g** Quantification of longitudinal muscle breaks. Discontinuation of longitudinal muscles in one layer of VM in the area captured with 40× objective (388 µm × 388 µm) was counted. *N* = 37 (*esg*^*ts*^), *N* = 19 (*esg*^*ts*^ > *Raf*^*gof*^), *N* = 21 (day 1, *esg*^*ts*^ > *Ras*^*V12*^) biological replicates. Mean ± SEMs are shown with individual data points. Data were analyzed by two-tailed unpaired Student’s *t*-test. Asterisks indicate statistical significance (*P < 0.01), and *P* values are indicated in graph, transgenes were induced with *esg*^*ts*^ for 2 days at 29 °C. In the side views of (**a**–**d**), the basal side of epithelia is positioned upward.

Actin-based cellular structures play crucial roles in invasive cell behaviors. In particular, invadopodia, which are actin- and cortactin-rich protrusions associated with degradation of the ECM^27,28^, are known to be essential for cancer-cell invasive phenotypes^28,29^. To address whether *Ras*^*V12*^ cells are forming similar invasive structures, we decided to elucidate actin cytoskeleton organization. Staining actin with phalloidin did not yield a discernable signal in ISCs and EBs because the adjacent VM expressed a large quantity of actin. Thus, we assessed Actin cytoskeleton organization by expressing actin-mRFP (*UAS-Actin-mRFP*) with *esg*^*ts*^. Intriguingly, actin-mRFP was detected as puncta at the basal side of *Ras*^*V12*^ cells that protruded toward the VM while similar puncta were undetectable in control cells (Fig. 3a, b). Of importance, these actin-rich protrusions frequently reached the outer surface by passing through the BM and VM layers (Fig. 3b). Furthermore, these protrusions were also detected as large blebs at day 2 of *Ras*^*V12*^ expression (Fig. 3c).

In cancer cells, invadopodia are detected as protrusions enriched for both actin and cortactin—a regulator of actin polymerization^28^. Since the shape of these protrusions observed in *Ras*^*V12*^ cells resembles that of invadopodia^28^, we examined cortactin localization by expressing HA-tagged cortactin (*UAS-Cortactin-HA*) with *esg*^*ts*^. We found that cortactin-HA was also detected as protrusions and large blebs at the basal side of *Ras*^*V12*^ cells (Supplementary Fig. 4b, c). In control cells, cortactin-HA was broadly detected at the cortical region and did not form prominent puncta or protrusions at the basal side (Supplementary Fig. 4a). To further confirm that cortactin-rich protrusions observed in *Ras*^*V12*^ cells were not formed due to an overexpression artifact, we expressed cortactin-HA in *Raf*^*gof*^ cells. Nevertheless, we found that cortactin-HA overexpression alone was not sufficient to induce similar protrusions and large blebs at the basal side of *Raf*^*gof*^ cells (Supplementary Fig. 4e, f). Notably, these cortactin-rich protrusions found in *Ras*^*V12*^ cells were also stained with phalloidin (Fig. 3d, arrowhead), indicating that these protrusions were enriched for both cortactin and actin.

Remarkably, these protrusions could even vertically penetrate the VM (Fig. 3d), resulting in damage to the tissue. In particular, we observed that the circular muscles around *Ras*^*V12*^ cells became torn and thinned (Fig. 3a–c; phalloidin staining). Interestingly, we detected large ruptures in the VM layer where multiple projections are concentrated (Fig. 3e and Supplementary Fig. 4d). When the midguts were viewed from the outside, a severe damage in VM was detected at day 2 of *Ras*^*V12*^ expression, which was manifested by breakage of the longitudinal muscles that normally span the whole posterior part of the midgut (Fig. 3f, g). We also noticed that a significant number of large blebs were visible outside of the posterior *esg*^*ts*^ > *Ras*^*V12*^ midguts (Fig. 3f, arrowhead). Altogether, these observations indicate that actin- and cortactin-rich protrusions are associated with the breach of the VM layer.

In cancer cells, cortactin plays a crucial role in formation of invadopodia as well as secretion of MMPs^30^. Knockdown of *cortactin* in cancer cells decreased MMP secretion and impaired ability to degrade the ECM by invadopodia^31^. Given the strong enrichment of cortactin in the protrusions detected in *Ras*^*V12*^ cells, we decided to deplete *cortactin* to gain more insight into the role of these protrusions. *Cortactin* knockdown in *Ras*^*V12*^ cells suppressed ECM degradation (Supplementary Fig. 5a, b) and rescued the muscle damage phenotype caused by *Ras*^*V12*^ cells (Supplementary Fig. 5c). Notably, cortactin depletion caused a slight, but significant reduction in Mmp1 levels (Supplementary Fig. 5d, e). As a consequence, cell dissemination is almost completely suppressed by cortactin depletion in *Ras*^*V12*^ cells (Supplementary Fig. 5f). Altogether, these results demonstrate that these actin- and cortactin-rich protrusions observed in *Ras*^*V12*^ cells are linked to the breach of the ECM as well as the VM layer.

Note that we detected Lifeact-mRFP also forming puncta at the basal side of *Ras*^*V12*^ cells (Supplementary Fig. 6). In general, these puncta marked with Lifeact-mRFP remained underneath the VM layer and did not grow into large protrusions, which might be explained by defect in actin caused by the overexpression of Lifeact^32^.

Our study describes the actin- and cortactin-rich protrusions that are associated with degradation of the ECM and the VM layer in *Drosophila*. The characteristics and the invasive nature of these protrusions resemble those of invadopodia observed in cancer cells^28^. Altogether, our observations demonstrate that dissemination of *Ras*^*V12*^ cells is an active process requiring cell-autonomous remodeling of cell morphology and formation of invasive protrusions at the basal side.

### *Ras*^*V12*^ cells produce large blebs and extracellular vesicles

**Fig. 4:**
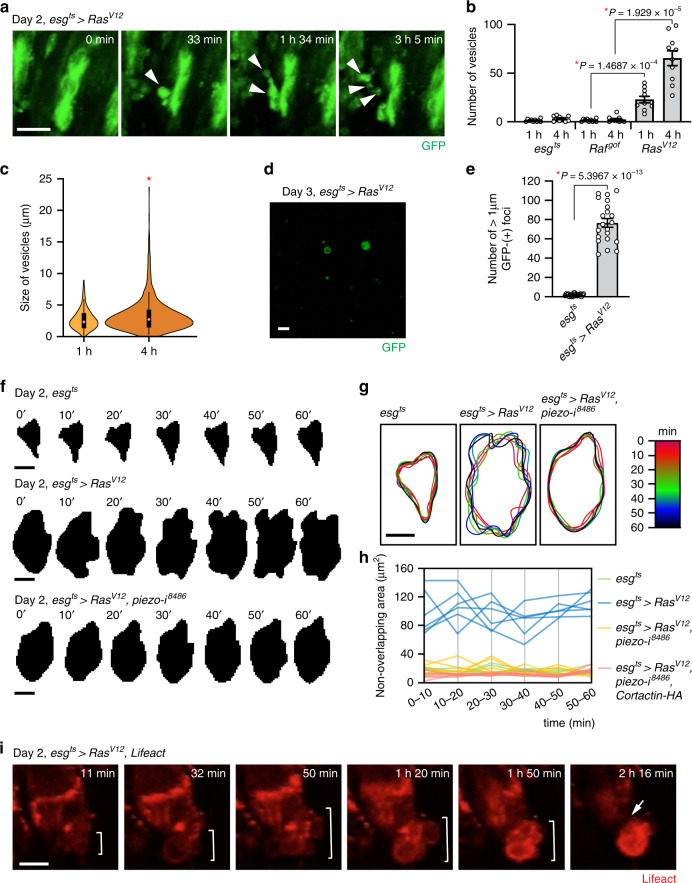
*Ras*^*V12*^ cells generate blebs and ELEVs and disseminate by extensive blebbing. **a** Still shots from ex vivo live imaging of *esg*^*ts*^ > *Ras*^*V12*^ midgut. Arrowheads indicate blebs and ELEVs. Scale bar, 10 µm. **b**, **c** Quantification of ELEVs generated from dissected midgut. Transgenes were induced for 2 days with *esg*^*ts*^. Number and size of ELEVs produced from dissected midgut were measured at 1 and 4 h post incubation in ex vivo live-imaging media. *N* = 10 biological replicates for each genotype. **b** Number of ELEVs. **c** Size of ELEVs. Circles show the medians (1 h, 3.0928 µm; 4 h, 3.6199 µm); box limits indicate the 25th and 75th percentiles; whiskers extend 1.5 times the interquartile range from the 25th and 75th percentiles. **d** Representative image of circulating ELEVs. Scale bar, 10 µm. **e** Quantification of GFP^+^ particles in hemolymph. Green particles bigger than 1 µm were counted. *N* = 21 from three independent experiments for each genotype. **f**–**h** Outline tracing of representative cells in *esg*^*ts*^, *esg*^*ts*^ > *Ras*^*V12*^, and *esg*^*ts*^ > *Ras*^*V12*^, *piezo RNAi*^*8486*^ midguts. **f** Time series of cell boundary masks. The representative cells were traced for an hour from ex vivo live-imaging videos. Masks of the cells were generated every 10 min. Scale bars, 10 µm. **g** Overlay of cell outlines. Time series of the cell silhouettes were color-coded and overlaid. Scale bar, 10 µm. **h** Deviation of cell boundary. Non-overlapping area of two cell silhouettes acquired at consecutive time points (*n*′ and *n* + 10′, *n* = 0′, 10′, 20′, 30′, 40′, 50′) is measured. *N* = 6 for each genotype. **i** Still shots from ex vivo live imaging of *esg*^*ts*^ > *Ras*^*V12*^ midgut. Lifeact is expressed to visualize filamentous actin (red). Brackets indicate blebbing. Arrow points out the detachment of the blebbing cell from the midgut epithelium. Scale bar, 10 µm. In (**b**, **e**), mean ± SEMs are shown with individual data points. Data were analyzed by two-tailed unpaired Student’s *t*-test. Asterisks indicate statistical significance (*P < 0.01), and *P* values are indicated in graph.

To gain further insights into how midgut epithelial cells disseminate by passing through the VM layers, we performed ex vivo live imaging of midguts. We noticed that *Ras*^*V12*^ cells produced large blebs, which were also formed across the VM and eventually released as extracellular vesicles (Fig. 4a, b and Supplementary Movies 1–3). In contrast, *Raf*^*gof*^ cells did not produce a significant number of blebs or extracellular vesicles even though expression of *Raf*^*gof*^ induced comparable cell proliferation (Fig. 4b and Supplementary Movie 4). The average size of these vesicles was ~3.1 ± 1.5 µm (mean ± SD) (Fig. 4c). Thus, they are bigger than exosomes (<100 nm) and microvesicles (100–1000 nm). Since these vesicles have not been previously described in *Drosophila*, we refer them as extremely-large extracellular vesicles (ELEVs). In addition, GFP-positive particles bigger than 1 µm were detected in hemolymph prepared from flies expressing *Ras*^*V12*^ but not from controls or flies expressing *Raf*^*gof*^, indicating that ELEVs were also produced in vivo (Fig. 4d, e). Interestingly, these ELEVs are reminiscent of large extracellular vesicles, including large oncosomes and cytoplasts, which are also implicated in the invasive phenotypes of cancer cells^33–35^.

Ameboid movement, which is characterized by extensive blebbing, is used by cancer cells to migrate through holes in the matrix and move without support of adhesion^36–38^. Production of ELEVs implies that *Ras*^*V12*^ cells may utilize ameboid movement. Indeed, we observed that *Ras*^*V12*^ cells in the posterior midgut often extensively remodeled their cell shape. Tracing the outline of representative *Ras*^*V12*^ cells in ex vivo time-lapse live-imaging videos further visualized these dynamic remodeling events (Fig. 4f–h). Notably, we observed that *Ras*^*V12*^ cells could migrate out from the midgut involving extensive blebbing (Fig. 4i and Supplementary Movie 5). In addition, *Ras*^*V12*^ cells could also slip out of the tissue without significant blebbing through a region where a large quantity of ELEVs had been generated (Supplementary Fig. 7 and Supplementary Movie 6). These observations suggest that *Ras*^*V12*^ cells mainly use blebbing to migrate out from the midgut.

### Disruption of Piezo impairs dissemination of *Ras*^*V12*^ cells

Given the breach of the BM and VM layers, *Ras*^*V12*^ cells need to sense the cues associated with it and elicit appropriate cellular responses to successfully transmigrate into the hemocoel. Since degradation of the ECM and ruptures in the VM layers could substantially alter the biomechanical microenvironment, we looked into the role of the mechanosensitive cation channel Piezo in the cell dissemination process.

**Fig. 5:**
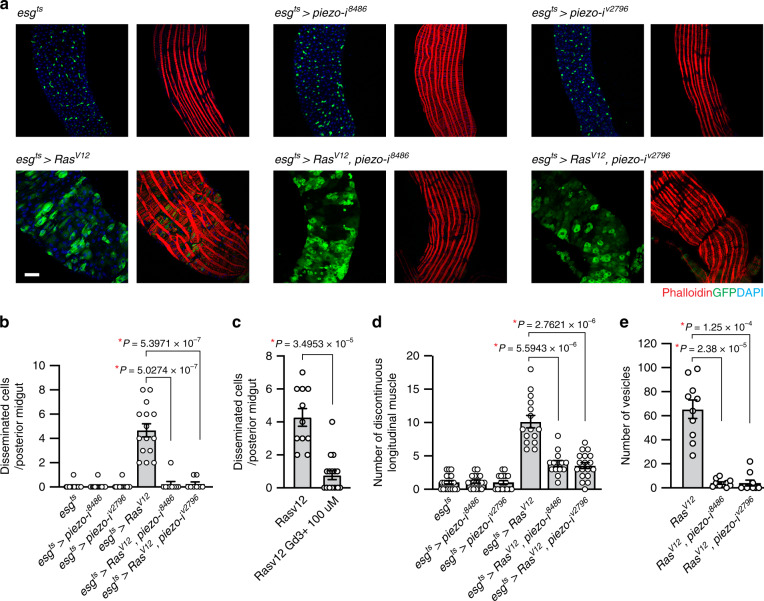
The mechanosensory channel Piezo is required for dissemination of *Ras*^*V12*^ cells. **a** Representative images of posterior midguts. Transgenes were induced for 2 days with *esg*^*ts*^. Scale bar, 50 µm. **b** Quantification of disseminated cells residing on the surface of VM. *N* = 20 (*esg*^*ts*^), *N* = 15 (*esg*^*ts*^ > *piezo-i*^*8488*^), *N* = 14 (*esg*^*ts*^ > *piezo-i*^*v2796*^), *N* = 15 (*esg*^*ts*^ > *Ras*^*V12*^), *N* = 9 (*esg*^*ts*^ > *Ras*^*V12*^*, piezo-i*^*8488*^), *N* = 8 (*esg*^*ts*^ > *Ras*^*V12*^*, piezo-i*^*v2796*^) biological replicates. **c** Quantification of disseminated cells detected on the surface of VM. 100 µM GdCl_3_-supplemented food was fed for 2 days while expressing *Ras*^*V12*^ with *esg*^*ts*^. *N* = 11 (*esg*^*ts*^ > *Ras*^*V12*^), *N* = 17 (*esg*^*ts*^ > *Ras*^*V12*^) biological replicates. **d** Quantification of discontinuous longitudinal muscles. *N* = 21 (*esg*^*ts*^), *N* = 18 (*esg*^*ts*^ > *piezo-i*^*8488*^), *N* = 18 (*esg*^*ts*^ > *piezo-i*^*v2796*^), *N* = 15 (*esg*^*ts*^ > *Ras*^*V12*^), *N* = 14 (*esg*^*ts*^ > *Ras*^*V12*^*, piezo-i*^*8488*^), *N* = 18 (*esg*^*ts*^ > *Ras*^*V12*^*, piezo-i*^*v2796*^) biological replicates. **e** Quantification of vesicles. *N* = 10 biological replicates for each genotype. In (**b**–**e**), mean ± SEMs are shown with individual data points. Data were analyzed by two-tailed unpaired Student’s *t*-test, asterisks indicate statistical significance (*P < 0.01), and *P* values are indicated in graph.

Disruption of Piezo in *Ras*^*V12*^ cells using RNA inference (RNAi) lines (NIG#8486R-3 and VDRC#v2796)^39,40^ led to a significant suppression in cell dissemination (Fig. 5a, b). It has been shown that a blocker of mechanically-activated cationic currents, gadolinium also efficiently inhibits mouse Piezo1-induced current^41^. We found that feeding gadolinium was also sufficient to suppress dissemination of *Ras*^*V12*^ cells (Fig. 5c). This suppression could not be simply explained by a reduction in cell number since *piezo* knockdown did not decrease the number of ISCs nor the division of *Ras*^*V12*^ cells (Supplementary Fig. 8a–c). Similarly, the division of *Ras*^*V12*^ cells was not decreased by gadolinium feeding (Supplementary Fig. 8d).

A recent study has demonstrated that Piezo regulates differentiation of enteroendocrine (EE) cells in *Drosophila* midgut^42^. However, we found that the overall EE cell number remained unchanged in *esg*^*ts*^ > *Ras*^*V12*^ midgut, indicating that expression of *Ras*^*V12*^ did not facilitate production of EEs (Supplementary Fig. 8e–g). In addition, the effect of *piezo* knockdown in *Ras*^*V12*^ cells on EE cell population was negligible (Supplementary Fig. 8e–g). Thus, a possible change in EE cell population due to Piezo inhibition cannot account for the suppression of *Ras*^*V12*^ cell dissemination.

To determine which process requires Piezo, we scrutinized the cellular phenotypes caused by *piezo* knockdown. Interestingly, formation of invasive protrusions was not inhibited by *piezo* knockdown as Lifeact puncta were still detected at the basal side of *Ras*^*V12*^, *piezo RNAi* cells (Supplementary Fig. 9). In contrast, the muscle damage phenotype was rescued by *piezo* knockdown in *Ras*^*V12*^ cells, and the large blebs/protrusions detected outside the posterior midguts were almost invisible in the *esg*^*ts*^ > *Ras*^*V12*^, *piezo RNAi* posterior midgut (Fig. 5a, d). Furthermore, we found that *piezo* knockdown significantly suppressed ELEV production (Fig. 5e and Supplementary Movies 7, 8). Similarly, blebbing cells were no longer detected in the posterior *esg*^*ts*^ > *Ras*^*V12*^, *piezo RNAi* midguts; the boundary of *Ras*^*V12*^, *piezo RNAi* cells was not altered dramatically over an hour of tracing (Fig. 4f). Altogether, our characterization of the *piezo* knockdown phenotypes suggests that during dissemination of *Ras*^*V12*^ cells, Piezo impinges on two different processes: the breach of the VM layer and the induction of blebbing.

### Piezo plays two discrete roles in *Ras*^*V12*^ cell dissemination

**Fig. 6:**
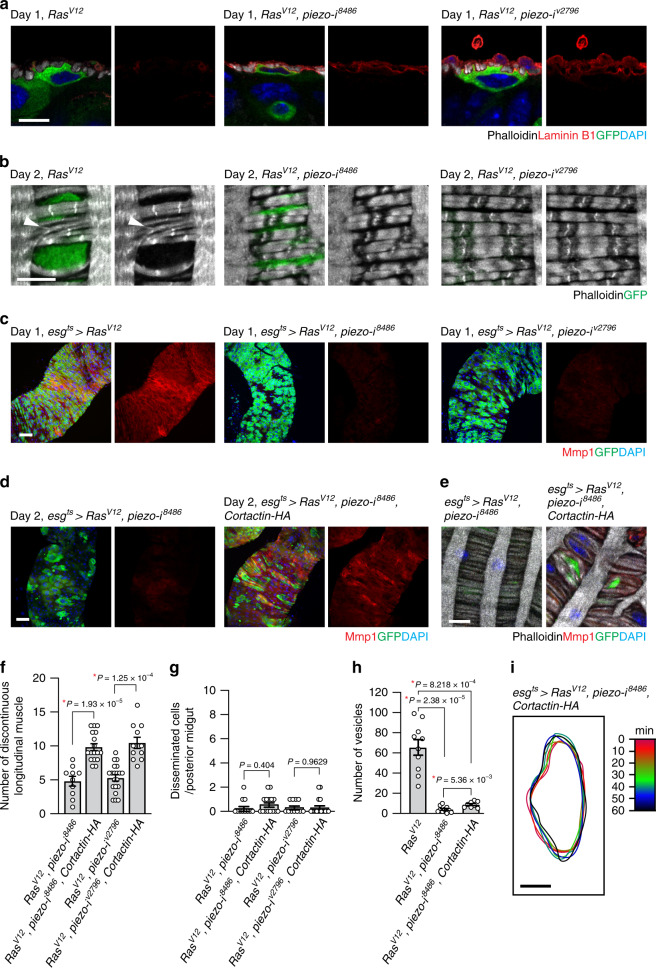
Piezo is required for induction of Mmp1 expression and blebbing. **a** Laminin B1 immunostaining (red). The basal side of epithelia is positioned upward. GFP and transgenes were induced for 1 day. Scale bar, 10 µm. **b** Magnified views of VM (gray). Arrowhead indicates torn circular muscles. Transgenes were expressed for 2 days. VM is visualized with phalloidin staining (gray). Scale bar, 10 µm. **c**, **d** Mmp1 staining (red) of posterior midguts. Scale bar, 50 µm. **e** Magnified views of VM (gray). Scale bar, 10 µm. **f** Quantification of longitudinal muscle break. *N* = 20 (*esg*^*ts*^ > *Ras*^*V12*^*, piezo-i*^*8488*^), *N* = 11 (*esg*^*ts*^ > *Ras*^*V12*^*, piezo-i*^*8488*^*, Cortactin-HA*), *N* = 10 (*esg*^*ts*^ > *Ras*^*V12*^*, piezo-i*^*v2796*^), *N* = 17 (*esg*^*ts*^ > *Ras*^*V12*^*, piezo-i*^*v2796*^*, Cortactin-HA*) biological replicates. **g** Disseminated cells detected outside VM. *N* = 11 (*esg*^*ts*^ > *Ras*^*V12*^*, piezo-i*^*8488*^), *N* = 17 (*esg*^*ts*^ > *Ras*^*V12*^*, piezo-i*^*8488*^*, Cortactin-HA*), *N* = 11 (*esg*^*ts*^ > *Ras*^*V12*^*, piezo-i* ^*v2796*^), *N* = 15 (*esg*^*ts*^ > *Ras*^*V12*^*, piezo-i*^*v2796*^*, Cortactin-HA*) biological replicates. **h** Quantification of vesicles at 4 h post incubation. The *Ras*^*V12*^ and *Ras*^*V12*^*, piezo-i*^*8486*^ quantifications were adapted from Fig. 5e for comparison. *N* = 10 (*esg*^*ts*^ > *Ras*^*V12*^), *N* = 10 (*esg*^*ts*^ > *Ras*^*V12*^*, piezo-i*^*8488*^), *N* = 8 (*esg*^*ts*^ > *Ras*^*V12*^*, piezo-i*^*8488*^*, Cortactin-HA*) biological replicates. **i** Overlay of cell outlines. A *Ras*^*V12*^, *piezo RNAi, Cortactin-HA* cell in ex vivo live-imaging video was traced for an hour. The cell’s silhouettes obtained every 10 min were overlaid. GFP and other transgenes were induced with *esg*^*ts*^ for 2 days. Scale bar, 10 µm. In (**f**–**h**), mean ± SEMs are shown with individual data points. Data were analyzed by two-tailed unpaired Student’s *t*-test. Asterisks indicate statistical significance (*P < 0.01), and *P* values are indicated in graph.

Our observations indicate that actin- and cortactin-rich invasive protrusions were associated with the breach of the ECM and the VM layer. Given the rescue of muscle damage by *piezo* depletion in *Ras*^*V12*^ cells, Piezo might regulate the function of these invasive protrusions. Since we found that *Cortactin* knockdown in *Ras*^*V12*^ cells suppressed the degradation of the ECM (Supplementary Fig. 5a,b), we tested whether *piezo* knockdown also rescued ECM degradation. Stainings of laminin B1 showed that Piezo was required for degradation of the ECM by *Ras*^*V12*^ cells (Fig. 6a). Furthermore, we found that *piezo* knockdown rescued the circular muscle thinning and segregation phenotype caused by *Ras*^*V12*^ expression (Fig. 6b). Of importance, *piezo* knockdown in *Ras*^*V12*^ cells greatly reduced Mmp1 levels (Fig. 6c), indicating that Mmp1 expression in *Ras*^*V12*^ cells was dependent upon Piezo. Multiple studies have shown that the calcium-dependent proteases calpains are critical mediators of calcium signaling induced by Piezo channels^43,44^. To elucidate the role of calpain in Mmp1 induction, we depleted *Drosophila Calpain A* (*CalpA*) and *Calpain-B* (*CalpB*) in *Ras*^*V12*^ cells, respectively, by RNAi approach. We found that knockdown of either *CalpA* or *CalpB* in *Ras*^*V12*^ cells significantly reduced Mmp1 levels (Supplementary Fig. 10a, b). In addition, *Calpain* knockdown in *Ras*^*V12*^ cells rescued the muscle damage phenotype (Supplementary Fig. 10a). Of importance, dissemination of *Ras*^*V12*^ cells was almost completely inhibited by depletion of either *CalpA* or *CalpB* (Supplementary Fig. 10c). Altogether, these results suggest that Piezo is a critical determinant of Mmp1 levels, and calpains might be a mediator of Piezo-induced calcium signaling in *Ras*^*V12*^ cells.

Piezo disruption induces additional distinct phenotypes: reduction in ELEVs and impairment in blebbing. If the molecular function of Piezo during cell dissemination can be attributed solely to the control of Mmp1 expression, the cell dissemination defect caused by *piezo* knockdown could be rescued by promoting Mmp1 expression. Cortactin is a key regulator of invadopodia assembly and function in cancer cells, and amplification of *cortactin* is linked to an increase in metastasis of carcinoma^31,45^. Furthermore, it has been shown that cortactin overexpression promotes invadopodia function and MMP activity in many types of cancer cells^31^. Given the conservation of the molecular function of cortactin in the control of actin assembly, we hypothesized that cortactin overexpression might rescue the cell dissemination defect caused by *piezo* knockdown by enhancing the invasive protrusion function. We found that cortactin overexpression in *Ras*^*V12*^*, piezo RNAi* cells restored Mmp1 levels (Fig. 6d), the formation of large blebs/protrusions across VM (Fig. 6e), and the VM damage (Fig. 6f) to the extent caused by *Ras*^*V12*^ cells. These results suggest that augmentation of cortactin levels in *Ras*^*V12*^ cells was sufficient to promote Mmp1 expression and compromise the integrity of the tissue. Nevertheless, it could not rescue the defect in dissemination of *Ras*^*V12*^*, piezo RNAi* cells (Fig. 6g), indicating that creating ruptures in the BM and VM layers was not sufficient to induce dissemination of *Ras*^*V12*^*, piezo RNAi* cells. Of significance, cortactin overexpression failed to restore ELEV production to the levels of *Ras*^*V12*^ cells even though it could slightly increase ELEV formation from *Ras*^*V12*^*, piezo RNAi* cells (Fig. 6h). Furthermore, *Ras*^*V12*^*, piezo RNAi* cells failed to produce blebs despite of ectopic cortactin expression (Fig. 6i and Supplementary Movie 9). Time-lapse outline tracing of representative cells in the tissue demonstrated that *Ras*^*V12*^*, piezo RNAi* cells with ectopic cortactin expression still remained steady and did not exert extensive blebbing (Figs. 4h and 6i). Altogether, these results suggest that Piezo plays a discrete role in eliciting blebbing, which is a way to transmigrate into the hemocoel.

## Discussion

**Fig. 7:**
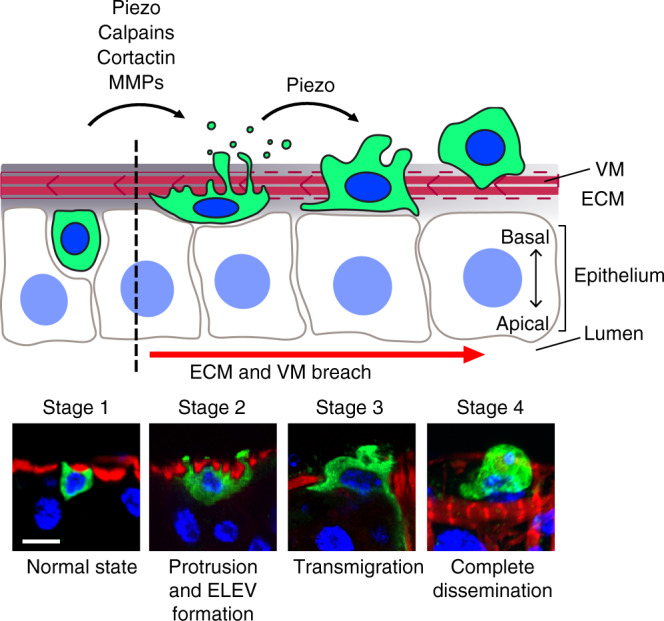
Schematic stages of cell dissemination. Stages of cell dissemination were reconstituted based on confocal images of fixed *Ras*^*V12*^ cells and ex vivo live imaging of *esg*^*ts*^ > *Ras*^*V12*^ posterior midguts. Lower panels show representative confocal images of control (*esg*^*ts*^; stage 1) and *Ras*^*V12*^ cells (stages 2–4). An *esg*^*ts*^ cell is illustrated in stage 1. In the absence of *Ras*^*V12*^ expression, ISCs and EBs reside within the midgut epithelia. Upon *Ras*^*V12*^ expression, invasive protrusions are formed at the basal side of the cells, and Mmp1 levels increase. Stage 2 illustrates a *Ras*^*V12*^ cell producing large protrusions/blebs and ELEVs across the VM. These cells could be observed at days 2 and 3 of *Ras*^*V12*^ expression. Stages 3 and 4 illustrate *Ras*^*V12*^ cells under and after transmigration, respectively. Disseminated cells were frequently detected at days 2 and 3 of *Ras*^*V12*^ expression. Scale bar, 10 µm.

Although previous studies have demonstrated that *Drosophila* transformed cells can metastasize to distant tissues^8,12,14,16,17^, it has been largely unknown how these transformed cells migrate from their primary site into the hemocoel. Here, we define a series of cellular processes and molecular mechanisms required for cell dissemination in *Drosophila* (Fig. 7). Initially, expression of *Ras*^*V12*^ in ISCs and EBs makes them propagate. These *Ras*^*V12*^ cells produce actin- and cortactin-rich invasive protrusions at the basal side, which grow into large blebs/protrusions penetrating the BM and VM layers (Fig. 7, stage 2). *Ras*^*V12*^ cells release ELEVs across the VM layer presumably from where these large blebs/protrusions are formed. Finally, *Ras*^*V12*^ cells transverse the VM layer by involving extensive blebbing or simply slipping through where tissue integrity is compromised (Fig. 7, stage 3) and complete the transmigration process (Fig. 7, stage 4). Note that we did not detect metastasis of *Ras*^*V12*^ cells although they were found in hemolymph. To metastasize, these cells might require additional genetic alterations to evade anoikis in hemolymph and reactivate cell division in distant tissues, avenues for further investigation. Altogether, our observations demonstrate how multiple invasive and migratory mechanisms are incorporated in vivo to form a mode of cell dissemination.

In this study, we describe the actin- and cortactin-rich invasive protrusions in *Drosophila*. Our observations show the striking resemblance between these invasive protrusions and invadopodia observed in cancer cells. In particular, our findings indicate that these invasive protrusions in *Ras*^*V12*^ cells are associated with the breach of the ECM. In cancer cells, disruption of cortactin, which is a major component in invadopodia, impaired invadopodia-mediated ECM degradation^31,30^. Similarly, cortactin was enriched at the invasive protrusions, and cortactin depletion in *Ras*^*V12*^ cells significantly suppressed the ECM degradation (Supplementary Fig. 5a,b) and dissemination of *Ras*^*V12*^ cells (Supplementary Fig. 5f). These results suggest that the invasive protrusions in *Drosophila* are functionally orthologous to invadopodia observed in cancer cells. Therefore, testing localization of additional invadopodium markers, such as tyrosine kinase substrate with five SH3 domains (Tks5), and measuring the release of MMPs at the invasive protrusions will help us to further highlight the resemblance between two invasive protrusions. Interestingly, a robust ECM degradation is a phenotype commonly observed in metastatic tumors in *Drosophila*^12–14,16,24^. However, it is unknown whether other transformed *Drosophila* cells also utilize similar invasive protrusions for cell dissemination. Therefore, it would be interesting to address other metastatic cells in *Drosophila*, such as *Ras*^*V12*^*/scrib*^*−/−*^ eye disc tumor cells^12^ and *Ras*^*V12*^-expressing hindgut epithelial cells^14^, utilize a similar invasive structure for cell dissemination.

Our observations suggest that the breach of the ECM and the VM layer associated with the action of the invasive protrusions could be exploited for cell dissemination. *Ras*^*V12*^ cells transmigrate into the hemocoel through the ruptures in the VM layer by employing extensive blebbing. In addition, *Ras*^*V12*^ cells can even slide out from the midgut through the regions that produce ELEVs. Notably, our observations show that some of these disseminated cells are not originally located near the dissemination site (Supplementary Movie 8). Altogether, these observations suggest that the breach of the ECM and VM layers provides an opportunity that nearby cells can utilize for dissemination. Nevertheless, our observations also demonstrate that this opportunity cannot be utilized when Piezo is disrupted (Fig. 6d–i). Given the extensive degradation of the ECM, mesenchymal mode of migration is not a proper migratory strategy that *Ras*^*V12*^ cells can adopt for dissemination since it requires formation of strong adhesions at the leading edge^37,46^. Multiple mechanisms that cells use to migrate without support of focal adhesions have been proposed^38^. In particular, bleb-driven ameboid movement allows cells to pass through holes in the three-dimensional substrate. Interestingly, we found that *Ras*^*V12*^ cells generate a large amount of ELEVs and blebs, suggesting that *Ras*^*V12*^ cells adopt bleb-driven ameboid movement. Notably, Piezo depletion in *Ras*^*V12*^ cells impaired production of ELEVs and blebs. The breach of the BM and VM layers would cause a significant remodeling of the biomechanical microenvironment. Therefore, we propose that Piezo plays a key role in transducing the opportunistic cues associated with the compromise in the tissue integrity to allow *Ras*^*V12*^ cells to adapt appropriate migratory modes for dissemination. A recent study demonstrated that confinement and low adhesion could induce ameboid movement in various mammalian cells^47^, which highlighted how the mechanical cues in the substrates influences a cell’s migratory strategy. Interestingly, Srivastava et al. showed that pressure sensing via Piezo makes *Dictyostelium* to generate blebs instead of pseudopods for migration^48^. Thus, it would be interesting to address what environmental cues drive Piezo activation in the midguts for coordination of the cell’s dissemination process.

We elucidated a mode of cell dissemination and uncovered Piezo as a key player of cell dissemination in vivo. Piezo disruption interferes with specific cellular phenotypes observed over the course of cell dissemination, indicating that some of the morphologically distinct stages and cellular processes observed during cell dissemination are also genetically discriminable. Given the description of the molecular and cellular mechanisms during an actual cell dissemination process, our study underscores the usefulness of *Drosophila* in deciphering the genetic basis of these invasive mechanisms in a native context.

## Methods

### Fly genetics and husbandry

Fly crosses were raised in vials containing standard cornmeal-agar medium and incubated at 18 °C throughout development and adulthood. Three- to ten-day-old nonvirgin female flies were used for all experiments. Flies were shifted to 29 °C for 1- to 6-days prior to dissection. During incubation at 29 °C, flies were transferred onto fresh food every 2 days.

To manipulate intestinal stem cells (ISCs) and enteroblasts (EBs), we used *esg-GAL4, tub-GAL80*^*ts*^, *UAS-GFP* (referred as *esg*^*ts*^). The experimental procedures are described previously^49^. Strains obtained from the Bloomington *Drosophila* Stock Center (BDSC) are the followings: *UAS-piezo-GFP*/*TM6B* (#58773), *UAS-Ras*^*V12*^ (III) (#4847), *UAS-Raf*^*gof*^ (#2033), *UAS-p35* (#5073), *UAS-Lifeact-mRFP* (#58362), *UAS-Actin-mRFP* (#24778), *UAS-cortactin-HA* (#9368), *ey-GAL4* (#5534), and *UAS-EGFP* (#5430). We also used *UAS-Ras*^*V12*^ (II) (laboratory stock). Cell dissemination phenotypes induced by our laboratory *UAS-Ras*^*V12*^ (II) and BDSC *UAS-Ras*^*V12*^ (III) (#4847) alleles were comparable. For the experiments to check subcellular distribution of Piezo-GFP and cortactin-HA, we omitted *UAS-GFP* from *esg*^*ts*^.

We used two *piezo* RNAi lines: 8486R-3^39^ (shown as *UAS-piezo-i*^*8486*^) from the National Institute of Genetics, Japan (NIG-Fly; https://shigen.nig.ac.jp/fly/nigfly) and v2796^40^ (shown as *UAS-piezo-i*^*v2796*^) from the Vienna *Drosophila* Resource Center (VDRC; www.vdrc.at). Knockdown efficiency of the *piezo* RNAi lines was assessed by expressing each line with a *piezo* promoter trap line, *piezo-GAL4* (BDSC #78335)^40^. We extracted RNA samples from five adult female flies to measure *piezo* mRNA levels by qPCR using the primer sequences: 5′-TTGCTCGTTCAGTGAGCGTC-3′ and 5′-AGGCACTAGCCATTCGATGAT-3′. *piezo* transcript levels were reduced to ~43% by *UAS-piezo-i*^*8486*^ and ~61% by *UAS-piezo-i*^*v2796*^ compared with the levels in *piezo-GAL4* only.

For other knockdown experiments, we obtained from BDSC: *UAS-cortactin* RNAi (BDSC #32871), *UAS-Calpain-A* RNAi (BDSC #29455), and *UAS-Calpain-B* RNAi (BDSC #25963). We also used *UAS-Calpain A* RNAi (NIG #7563R-3) from NIG-Fly.

### Antibodies and immunofluorescence imaging

We used the following primary antibodies: anti-GFP antibody, Alexa Fluor® 488 (1:1000; Thermo Fisher Scientific, A-21311; rabbit), anti-Dl antibody (1:1000; Developmental Studies Hybridoma Bank, C594.9B; mouse), anti-Mmp1 antibody (1:1000; Developmental Studies Hybridoma Bank, 3B8D12; mouse), anti-phospho-histone H3 antibody (1:1000; Millipore, 06-570; rabbit), anti-phospho-histone H3 antibody (1:1000; Abcam, ab14955; mouse), anti-laminin B1 antibody (1:1000; Abcam, ab47650; rabbit), and anti-HA antibody (1:2000; Santa Cruz, SC7392; mouse). Secondary antibodies used in this study were anti-rabbit and anti-mouse IgGs conjugated to Alexa Flour® 594 or Alexa Fluor® 647 (1:1000; Thermo Fisher Scientific, A-11012, A11005, A-21244, A-21235; goat). Filamentous actin was stained with phalloidin conjugated to Alexa Fluor® 594 or 647 (1:1000; Thermo Fisher Scientific, A-12381, A-22287). Nuclei were stained with DAPI (1:2000; Sigma, D9542).

To remove food from the midguts, we fed flies on 4% sucrose for ~4 h prior to dissection. Female flies were dissected in PBS. The dissected midguts were fixed in 4% paraformaldehyde (PFA) (Electron Microscopy Sciences, RT15710) diluted in PBS for 20 min and then washed three times with PBST (PBS supplemented with 0.2% Triton X-100) for 5 min each. For permeabilization and blocking, we incubated the tissue samples in blocking buffer (PBST supplemented with 5% normal goat serum) for 1 h at room temperature. The tissue samples were incubated with primary antibodies in the blocking buffer either overnight at 4 °C or for 2–3 h at room temperature. The samples were washed three times with PBST and then incubated with secondary antibodies for 2–3 h at room temperature. Stained midguts were washed three times with PBST and mounted with Vectashield (Vector Laboratories, H-1000). Fluorescence micrographs were acquired with a Leica SP8 laser scanning confocal microscope with 20×/0.7 dry or 40×/1.25 oil objective lenses. Higher resolution images were acquired using Leica LIGHTNING—a detection package for image information extraction of confocal images. 3D reconstructions were created from z-stack images using a LAS X 3D viewer. NIH ImageJ software was used for further adjustment and assembly of the acquired images.

### Quantification of disseminated cells

We defined disseminated cells as GFP- and DAPI-positive cells residing more basally than the visceral muscle layer, which was labeled with phalloidin. To determine the position of a cell, we captured a series of z-stacks using confocal microscopy. Orthogonal view reconstituted from z-stacks was used for further confirmation. The number of disseminated cells was counted from the R5 region of the posterior midgut captured in 388 µm × 388 µm confocal microscope fields.

### Quantification of Mmp1 intensity

To measure the fluorescent intensity of Mmp1, a z-projection of 388 µm × 388 µm microscope field was created from the R5 region of a posterior midgut. The projection included stacks of one leaflet of the intestine. We collected mean gray value of the red channel from three random 100 µm × 100 µm fields per intestine using NIH ImageJ software and subtracted the background values measured from the outside area surrounding the intestine.

### Quantification of phospho-histone H3 (pHH3)-positive cells

To determine the number of dividing cells, midguts were dissected and stained with DAPI and anti-pHH3 antibody. pHH3-positive nuclei were counted from the entire midgut.

### Actin time-lapse imaging

To monitor filamentous actin during ex vivo live imaging, we expressed Lifeact-mRFP with *esg*^*ts*^. Five- to seven-day-old adult flies were incubated at 29 °C for 2 days before imaging. Time-lapse imaging of the posterior midgut was captured with a Leica SP8 laser scanning confocal microscope using a 40×/1.25 oil objective. Z-stacks of the dual-color images were recorded every 1 min for 2 h. A maximum intensity z-projection was obtained using Leica LAS X imaging software.

### Ex vivo live imaging

The experimental procedures described in ref. ^50^ were adapted for ex vivo live imaging of midguts. Our ex vivo live-imaging medium comprises Schneider’s *Drosophila* medium (Thermo Fisher Scientific, 21720024), 2% FBS (Life Technologies, 16140071), and 0.5% penicillin-streptomycin (Thermo Fisher Scientific, 15140122).

*Drosophila* were dissected in the imaging media and the entire midgut and hindgut sections were recovered. Then, the dissected samples were mounted on a 35-mm glass bottom dish (MatTek, P35G-1.0-14-C). To prevent squeezing, vacuum grease was applied as two lines on the glass bottom dish, ~2 mm apart. Approximately 100 µl of imaging media was added to the center of the dish, and the dissected guts were placed perpendicular to the vacuum grease lines. Anterior end of the midgut and hindgut segment were embedded in each vacuum grease line to prevent drifting. A cover glass was gently placed on top of the vacuum grease lines to prevent excessive movement of the midgut. Approximately 1 ml of imaging medium was added to the dish to prevent dehydration and facilitate gas exchange. To prevent media evaporation, the samples were covered with the dish lid and were imaged from the bottom side.

### Quantification of vesicles released from gut

Ex vivo live imaging of midguts was processed into z-projection videos with the Leica LAS X software. The number and diameter of the GFP-positive vesicles detected outside the midguts at the indicated time points were quantified using NIH ImageJ software.

### Collection of circulating ELEVs and disseminated cells

Hemolymph was collected through punctures on the head as described^51^. Hemolymph prepared from three flies was mixed with 3 μl of Vectashield with DAPI (1:2000) for immediate imaging. Seven random views of 500 µm × 500 µm confocal microscope fields were obtained with a Leica SP8 laser scanning confocal microscope with 40×/1.25 oil objective. ELEVs larger than 1 µm were counted using the NIH ImageJ analysis particle function. The number of circulating cells was counted manually by finding foci that were positive for both GFP and DAPI.

### Quantification of longitudinal muscle breakage

The number of discontinuous longitudinal muscle was counted from one leaflet of the R5 region of the posterior midgut captured in 388 µm × 388 µm confocal microscope fields. F-actin-rich visceral muscle (VM) fibers were revealed by phalloidin staining. Z-stack images were acquired using a Leica SP8 laser scanning confocal microscope with 40×/1.25 oil objective.

### Gd^3+^ feeding

Three- to seven-day-old adult flies were transferred onto standard media supplemented with either solvent (distilled water) or 100 µM GdCl_3_ (EMD Millipore, G7532-5G), then allowed to feed while the transgenes were induced at 29 °C.

### Cell outline tracing

We picked cells with distinguishable boundary and followed them for 1 h in ex vivo live-imaging z-projection videos. Still shots were captured every 10 min. At each timepoint, cell outline was traced using freehand area selection tool and subjected to create mask in NIH ImageJ software. To visualize the changes in cell boundary overtime, the traced cell outlines were overlaid. To quantify the changes in cell boundary, we measured non-overlapping area of two cell silhouettes acquired at consecutive time points (*n*′ and *n* + 10′, *n* = 0′, 10′, 20′, 30′, 40′, 50′).

### Statistics and reproducibility

All the images presented and used for quantification are from the posterior R5 region of adult female fly midguts, except for the pHH3, which is counted from the entire midgut. All experiments were independently repeated at least three times. Statistical analyses were performed using Microsoft Excel, GraphPad Prism 8, and ‘R’ software. All *P* values were determined by two-tailed Student’s *t*-test with unequal variances. Level of significance are depicted by asterisks in the figures: *P < 0.01. *P* values are indicated in graph. Sample sizes were chosen empirically based on the observed effects and listed in the figure legends.

### Reporting summary

Further information on research design is available in the Nature Research Reporting Summary linked to this article.

## Supplementary information

Supplementary Information

Description of Additional Supplementary Files

Supplementary Movie 1

Supplementary Movie 2

Supplementary Movie 3

Supplementary Movie 4

Supplementary Movie 5

Supplementary Movie 6

Supplementary Movie 7

Supplementary Movie 8

Supplementary Movie 9

Reporting Summary

## Data Availability

The authors declare that the data supporting the findings of this study are available within the paper and its supplementary information files. Additional datasets are available from the corresponding author upon reasonable request.
