# Supplementary material for: Dissemination of RasV12-transformed cells requires the mechanosensitive channel Piezo
Source: Nat Commun. 2020 Jul 16;11:3568. doi: 10.1038/s41467-020-17341-y (PMC7366633; doi:10.1038/s41467-020-17341-y)
Supplement: Supplementary file 1 — Supplementary Information [file 41467_2020_17341_MOESM1_ESM.pdf]

Supplementary Information

Dissemination of *Ras*^{V12}-transformed cells requires the mechanosensitive channel Piezo

Lee et al.

This PDF file contains:

- Supplementary Figures and Legends 1-10

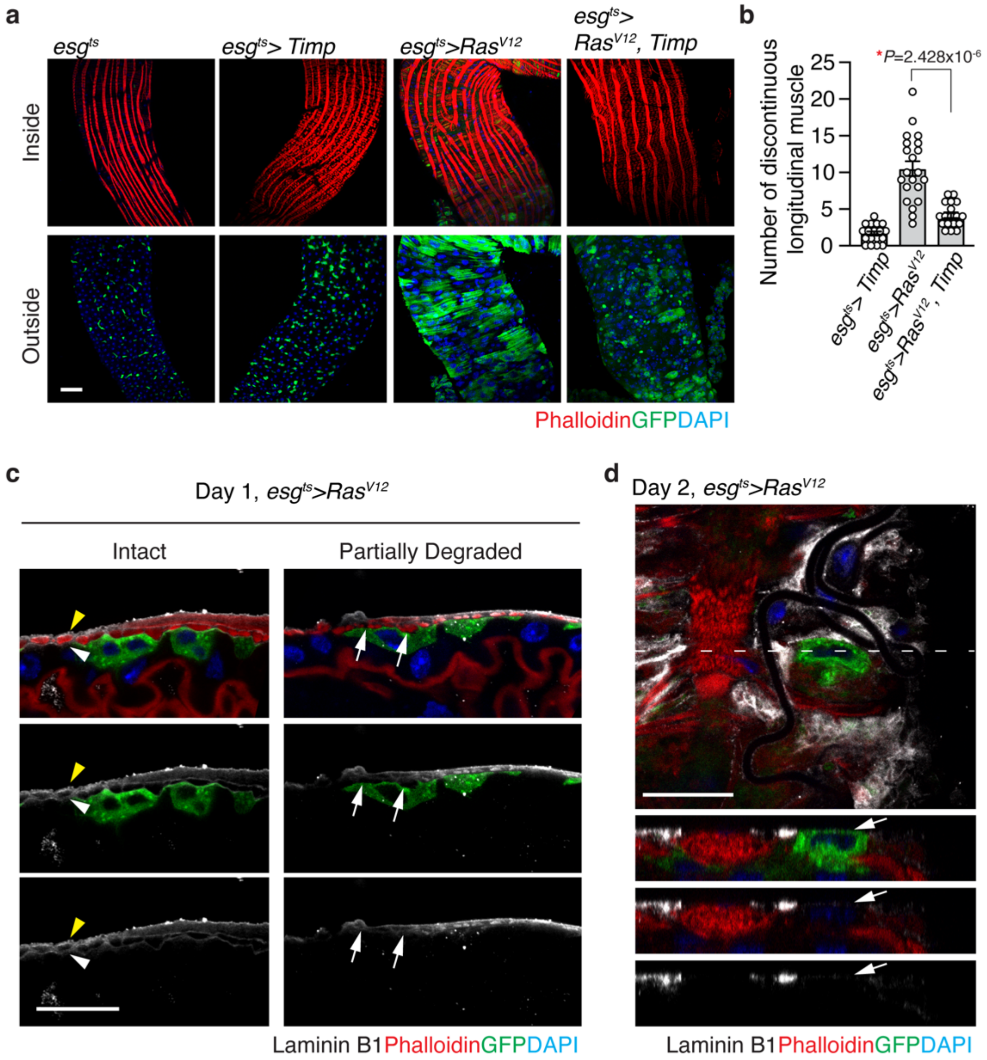

Supplementary Figure 1. Inhibition of Mmp suppresses dissemination of *Ras^{V12}* cells.

a, Representative images of the posterior midgut at day 2 of transgene expression. Scale bar, 50μm. **b,** Quantification of longitudinal muscle break. N=18 (*esg^{ts}>Timp*), N=21(*esg^{ts}>Ras^{V12}*), N=18 (*esg^{ts}>Ras^{V12}*, *Timp*) biological replicates. *Ras^{V12}* quantification is adopted from Fig. 3g. **c**, Laminin B1 staining from side view on day 1 *esg^{ts}>Ras^{V12}* midguts. White arrowhead points to the inner ECM layer adjacent to the epithelium, and yellow arrowhead points to the layer outside of the VM. Degradation of the inner ECM layer is pointed with white arrows. Scale bar, 20μm. **d**, Laminin B1 staining from top view on day 2 *esg^{ts}>Ras^{V12}* midguts. Arrow points to the basal side of a *Ras^{V12}* cell, indicating that the cell was not imbounded by a Laminin layer. Scale bar, 20μm. In the side views, the basal side of epithelium is positioned upward. In **b**, mean±SEMs are shown with individual data points. Data were analyzed by two-tailed unpaired Student's *t*-test. Asterisk indicates statistical significance (*P<0.01), and *P*-value is indicated in graph.

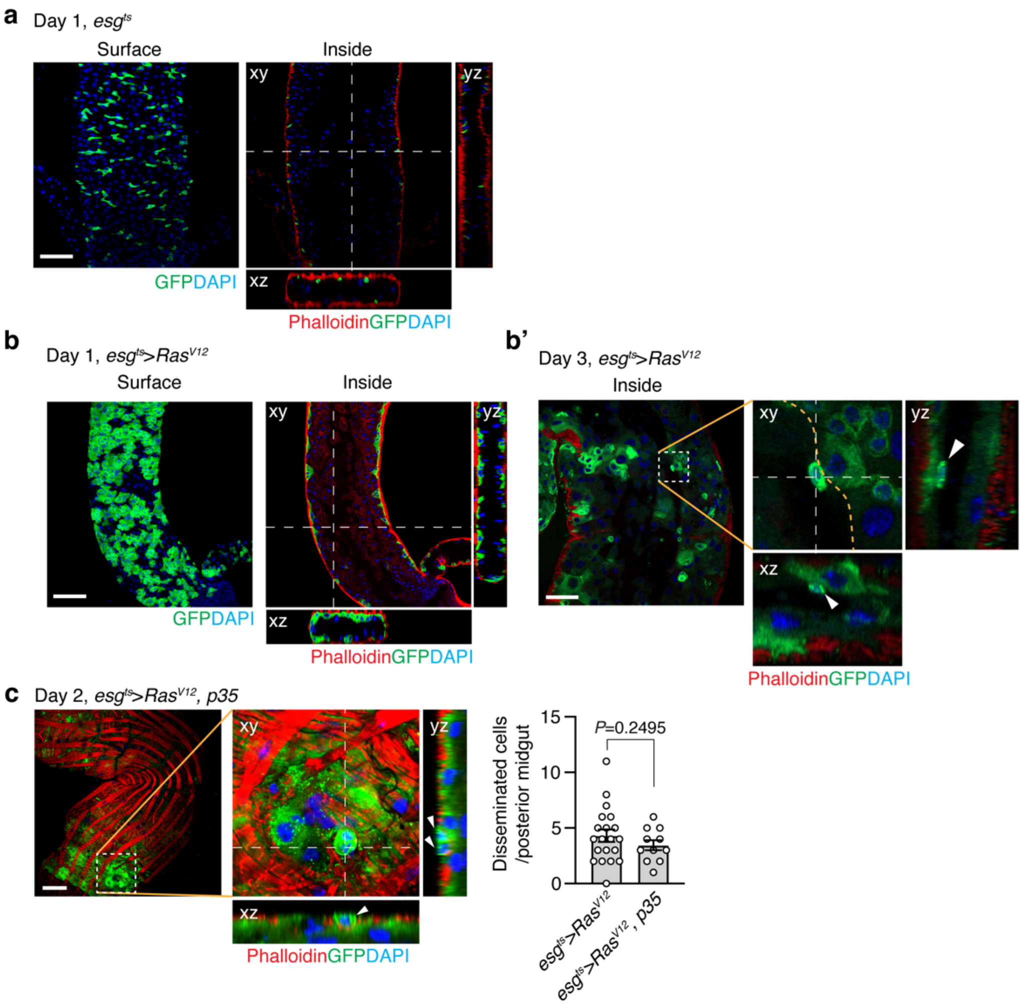

Supplementary Figure 2. Expression of *Ras*^{V12} also induces delamination into lumen.

a–b, Representative images of the posterior midguts at day 1 of transgene expression. At day 1, *Ras*^{V12} cells detached from the VM layer were rarely detected. **b'**, Representative image of the posterior *esg*^{ts}>*Ras*^{V12} midgut at day 3 of *Ras*^{V12} expression. *Ras*^{V12} cells delaminating into lumen were frequently detected at day 2 and 3 of *Ras*^{V12} expression. Inset shows *Ras*^{V12} cells detached from the VM layer and a *Ras*^{V12} cell (arrowhead) that appears to be shedding into lumen. Yellow dotted line indicates the apical boundary of the midgut epithelium. VM is visualized with Phalloidin (red), and nuclei are stained with DAPI (blue). Scale bars, 50μm. **c**, Representative image of the posterior *esg*^{ts}>*Ras*^{V12}, *p35* midgut. The anti-apoptotic protein p35 was expressed in *Ras*^{V12} cells with *esg*^{ts} for 2 days. Inset and the magnified views show basally disseminated cells (indicated with arrowheads). Scale bar, 50μm. Graph on right shows quantification of disseminated cells detected on the surface of the posterior midgut. N=20 (*esg*^{ts}>*Ras*^{V12}), N=11(*esg*^{ts}>*Ras*^{V12}, *p35*) biological replicates. Mean±SEMs are shown with individual data points. Data were analyzed by two-tailed unpaired Student's *t*-test. *P*-value is indicated in graph.

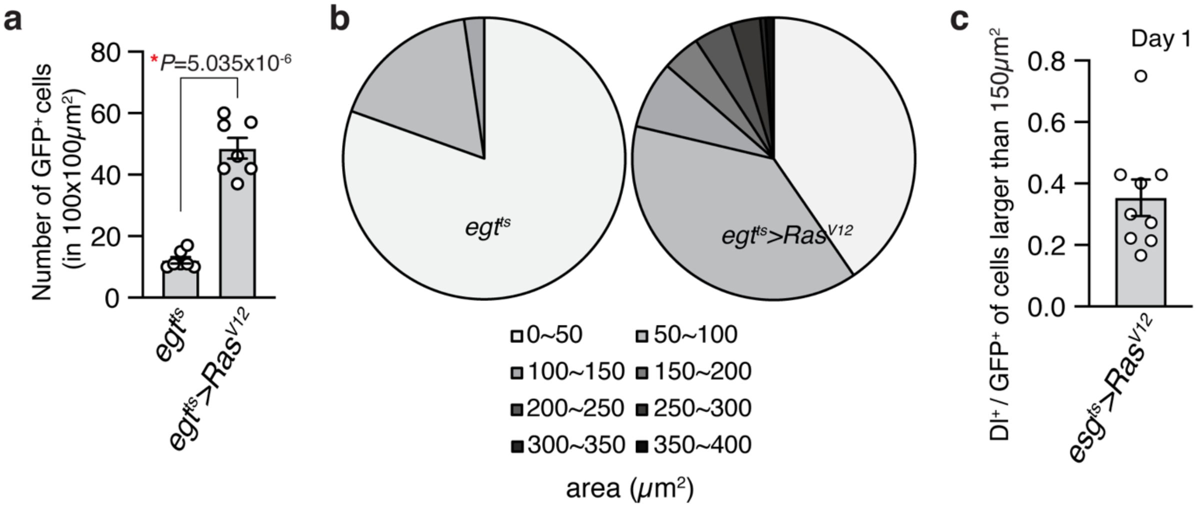

Supplementary Figure 3. *Ras^{V12}* cells undergo proliferation and enlargement.

a, Quantification of ISC and EB cells in the *esg^{ts}>Ras^{V12}* midgut at day 1 of transgene expression. Number of GFP^{+} cells were counted in 100x100 μm^{2} of the posterior midgut. N=11 biological replicates for each genotype. **b**, Quantification of the size of GFP^{+} cells at day 1. N=11 biological replicates for each genotype. **c,** Quantification of the proportion of Dl^{+} cells in *esg^{ts}>Ras^{V12}* midgut that are larger than normal *esg^{ts}* cells. *Ras^{V12}*-expressing cells that are both Dl^{+} positive and larger than 150 μm^{2} were counted and divided by the number of total GFP^{+} positive cells. N=9 biological replicates. In **a**, mean±SEMs are shown with individual data points. Data were analyzed by two-tailed unpaired Student's *t*-test. Asterisk indicates statistical significance (*P<0.01), and P-value is indicated in graph.

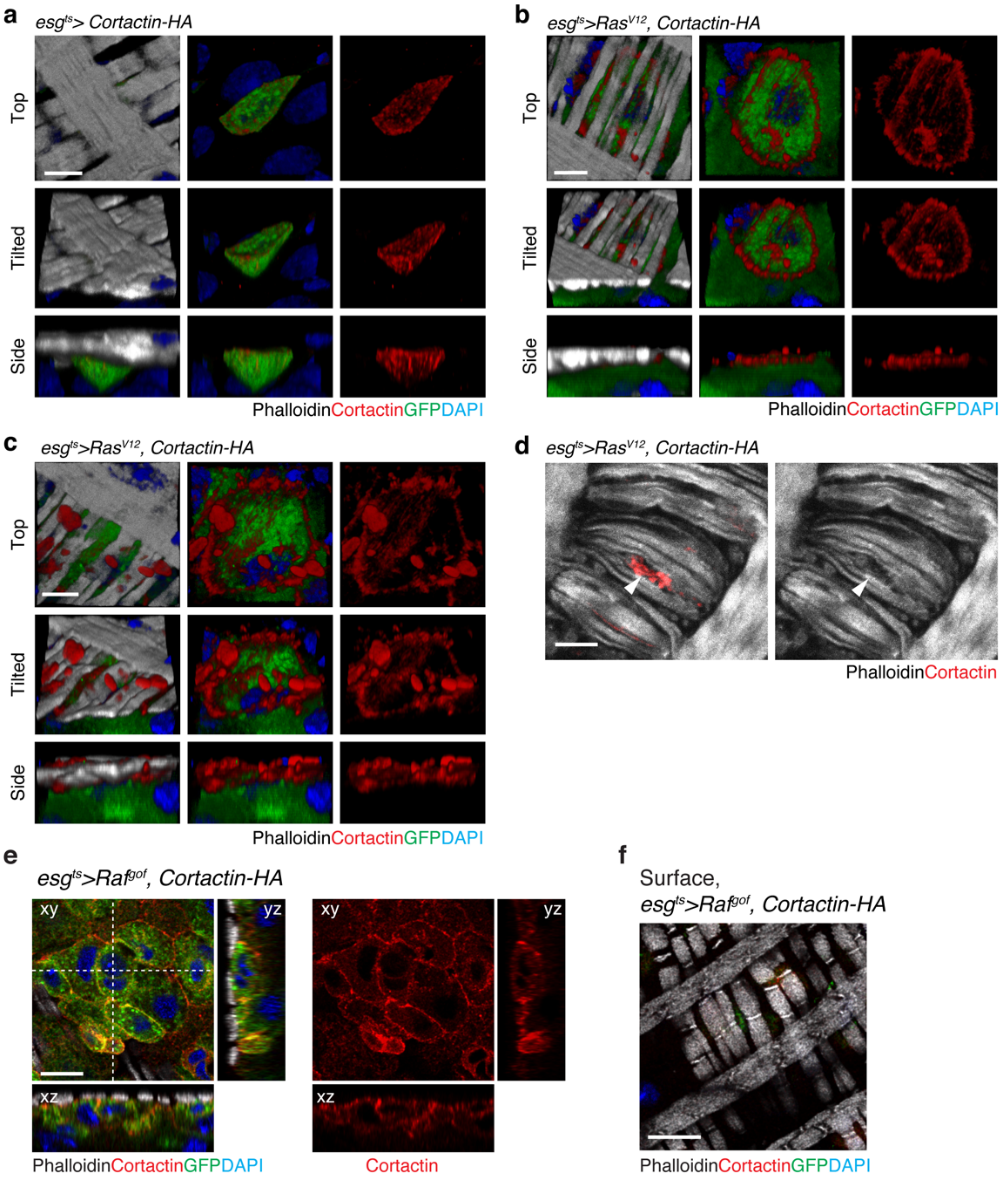

Supplementary Figure 4. Basal Cortactin-rich protrusions disrupt visceral muscle integrity.

a-c, 3D reconstructions of confocal images angled at 0, 45, and 90 degrees. Representative images of *esg*^{ts} cells (**a**) and *esg*^{ts}*>Ras*^{V12} cells (**b, c**) are shown. Dissected guts were stained with anti-HA antibody for Cortactin (red), Phalloidin (gray) for Actin, and DAPI (blue) for nuclei. Scale bars, 5μm. N=8 (*esg*^{ts} *>Cortactin-HA*) for **a** and N=20 (*esg*^{ts}*>Ras*^{V12}, *Cortactin-HA*) biological replicates for **b, c** and **d**. **d,** Surface view of a rupture in the VM layer associated with a cluster (arrowhead) of Cortactin-rich protrusions (red). Scale bar, 5μm. **e**-**f**, Top and orthogonal views (**e**) and surface view (**f**) of *esg*^{ts}*>Raf*^{gof}*, Cortactin-HA* cells at day 4 of transgene expression. Note that the basal Cortactin-rich protrusions were not formed by overexpression of Cortactin-HA in *esg*^{ts}*>Raf*^{gof} cells. Scale bars, 10μm. In the side views, the basal side of epithelium is positioned upward.

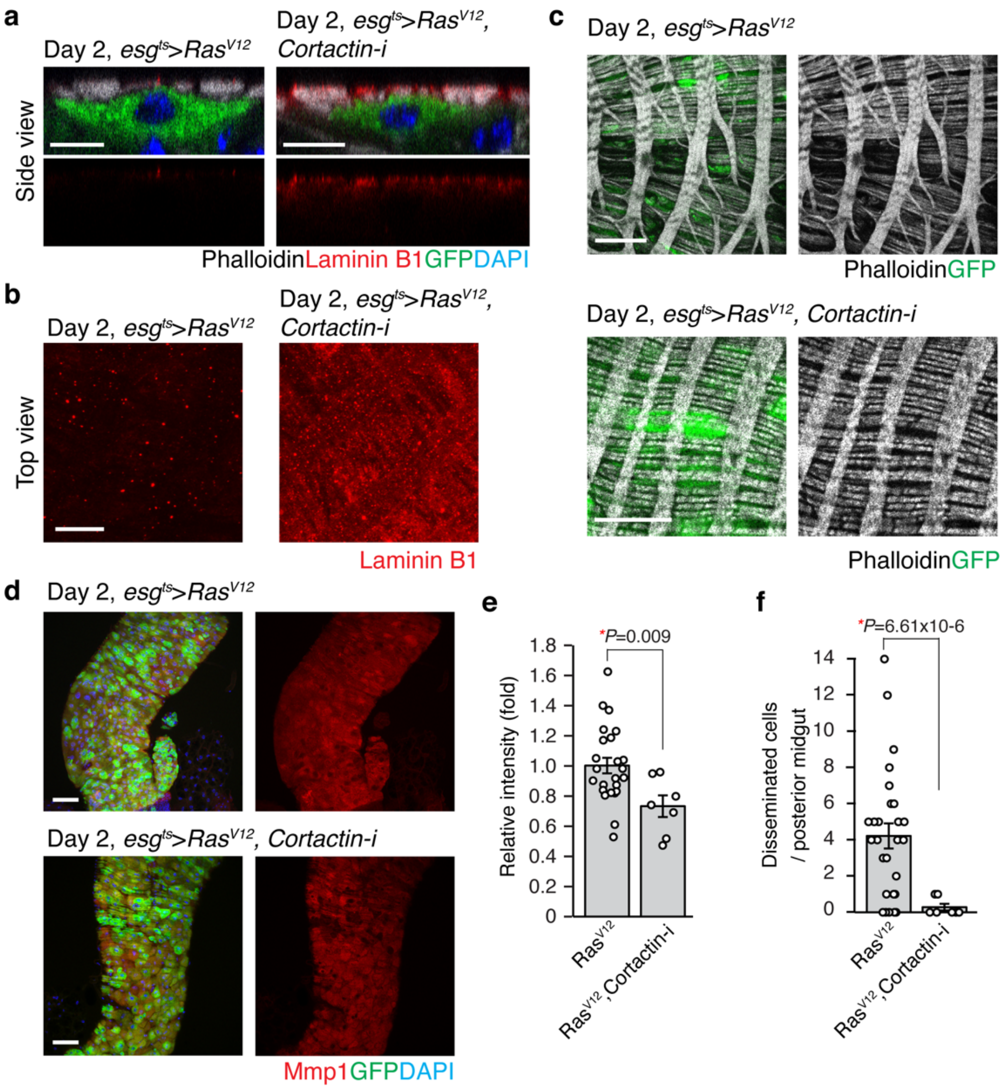

Supplementary Figure 5. *Cortactin* knockdown in *Ras^{V12}* cells suppresses Laminin degradation, Mmp1 expression, and cell dissemination.

a, Side view showing Laminin B1 expression. *esg^{ts}>Ras^{V12}* and *esg^{ts}>Ras^{V12}*, *Cortactin-i* midguts were stained with anti-Laminin B1 antibody (red), Phalloidin (gray), and DAPI (blue). Scale bars, 10*μ*m. **b,** Top view showing Laminin B1(red) expression. Scale bar, 10*μ*m. **c,** Visceral muscle (gray) integrity on day 2 of transgene expression. Scale bars, 30*μ*m. **d,** Mmp1 immunostaining (red) of posterior midguts. Scale bars, 50*μ*m. **e,** Quantification of Mmp1 levels per midgut: N=24 (*esg^{ts}>Ras^{V12}*), N=7 (*esg^{ts}>Ras^{V12}, Cortactin-i*) biological replicates. 3 regions were measured from each sample. **f,** Quantification of disseminated cells at day 2 of transgene expression: N=27 (*esg^{ts}>Ras^{V12}*), N=7 (*esg^{ts}>Ras^{V12}*, *Cortactin-i*) biological replicates. In **e** and **f**, mean±SEMs are shown with individual data points. Data were analyzed by two-tailed unpaired Student's *t*-test. Asterisks indicate statistical significance (*P<0.01), and *P*-values are indicated in graph.

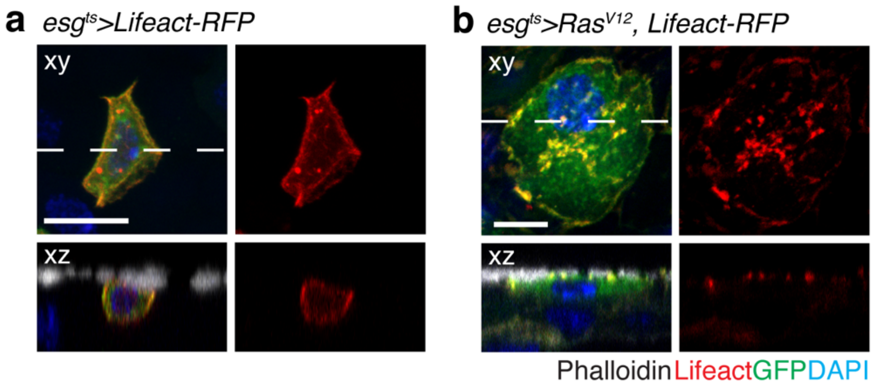

Supplementary Figure 6. F-actin-rich puncta are formed at the basal side of *Ras*^{V12} cells.

a, Lifeact (red) distribution in an *esg*^{ts} control cell. Scale bar, 10μm. **b**, Lifeact-rich puncta are formed at the basal side of an *esg*^{ts}>*Ras*^{V12} cell adjacent to the VM layer (Phalloidin, gray). Cells at day 2 of transgene expression are shown. Representative images are shown from N=11 (**a**) and N=16 (**b**) biological replicates. Scale bar, 10μm.

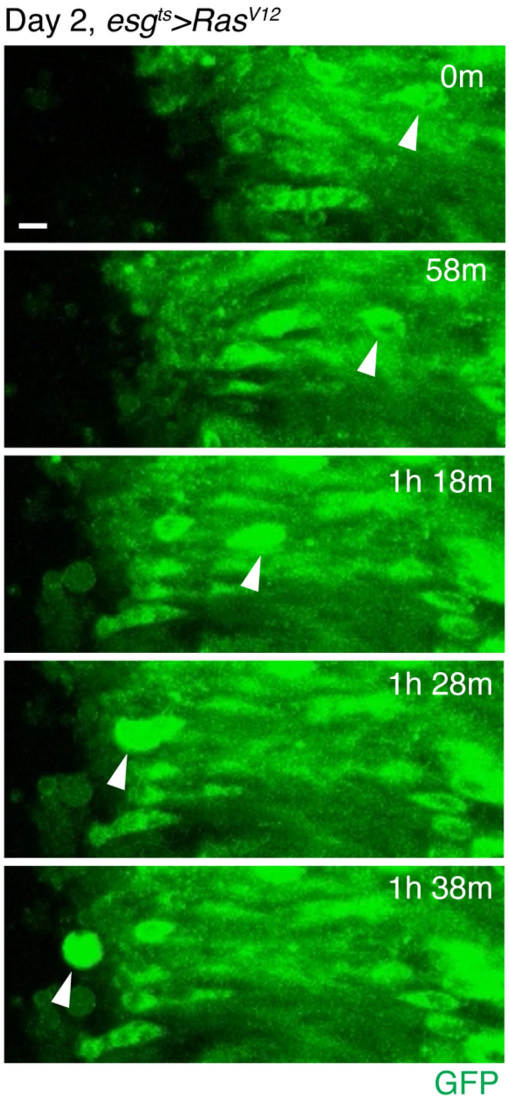

Supplementary Figure 7. Disseminating *Ras*^{V12} cell in a slipping motion.

Still shots from *ex vivo* live imaging of day 2 *esg*^{ts}>*Ras*^{V12} midgut (Supplementary Video 8). Arrowheads point to the disseminating cell at indicated time points. Scale bar, 10μm.

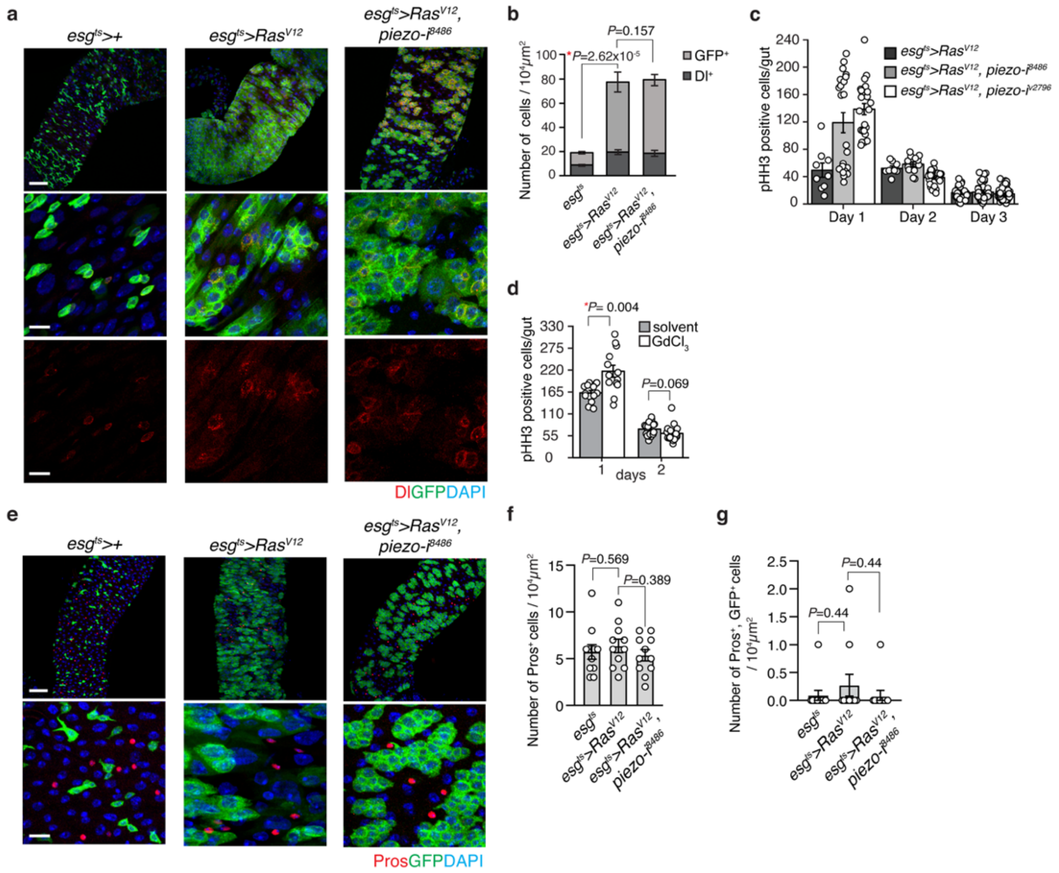

Supplementary Figure 8. Differentiation of EE cells are not affected by *piezo* knockdown in Ras^{V12} cells.

a, Representative image of the posterior midguts at day 1 of transgene expression. esg^{ts}, esg^{ts}>Ras^{V12}, and esg^{ts}>Ras^{V12}, $piezo\text{-}i^{8486}$ midguts were stained with anti-Dl antibody (red) and DAPI (blue) Scale bars, 50μm (top), 10μm (middle and bottom). **b**, Quantification of Dl^{+} and GFP^{+} cells at day 1 of transgene expression. N=11 biological replicates. **c,** Quantification of pHH3 cells per gut after 3 days of expressing two RNAi lines (VDRC#v2796 and NIG#8486R-3) to knockdown *piezo*. Day 1: N=9 (esg^{ts}>Ras^{V12}), N=22 (esg^{ts}>Ras^{V12}, $piezo\text{-}i^{8486}$), N=23 (esg^{ts}>Ras^{V12}, $piezo\text{-}i^{v2796}$); Day 2: N=8 (esg^{ts}>Ras^{V12}), N=13 (esg^{ts}>Ras^{V12}, $piezo\text{-}i^{8486}$), N=23 (esg^{ts}>Ras^{V12}, $piezo\text{-}i^{v2796}$); Day 3: N=21 (esg^{ts}>Ras^{V12}), N=35 (esg^{ts}>Ras^{V12}, $piezo\text{-}i^{8486}$), N=41 (esg^{ts}>Ras^{V12}, $piezo\text{-}i^{v2796}$) biological replicates. **d**, Quantification of pHH3 positive cells. Gd^{3+} treatment does not decrease cell proliferation. Day 1: N=14 (solvent), N=13 ($GdCl_3$); Day 2: N=19 (solvent), N=20 ($GdCl_3$) biological replicates. **e,** Representative image of the posterior midguts. EE cells were visualized by staining with anti-Prospero antibody (red). Scale bars, 50μm (top), 10μm (bottom). **f,** Quantification of Pros^{+} cells at day 1 of transgene expression. N=11 biological replicates. **g,** Quantification of Pros^{+} and GFP^{+} cells at day 1 of transgene expression. N=11 biological replicates. In **b**, **c**, **d,** **f**, and **g**, mean±SEMs are shown with individual data points. Data were analyzed by two-tailed unpaired Student's *t*-test. Asterisks indicate statistical significance (*P<0.01), and *P*-values are indicated in graph.

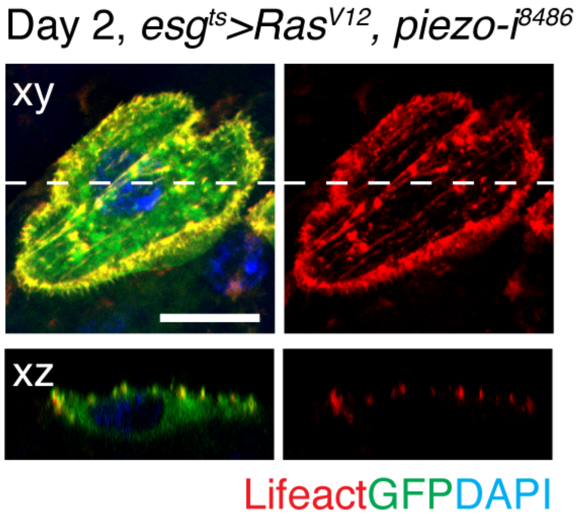

Supplementary Figure 9. ***Piezo*** **is dispensable for Actin-rich puncta formation.**

Lifeact puncta (red) were detected at the basal side of the *Ras*^{V12}*, piezo* RNAi cells at day 2 of transgene expression. Representative images are shown from N=13 biological replicates. Scale bar, 10μm.

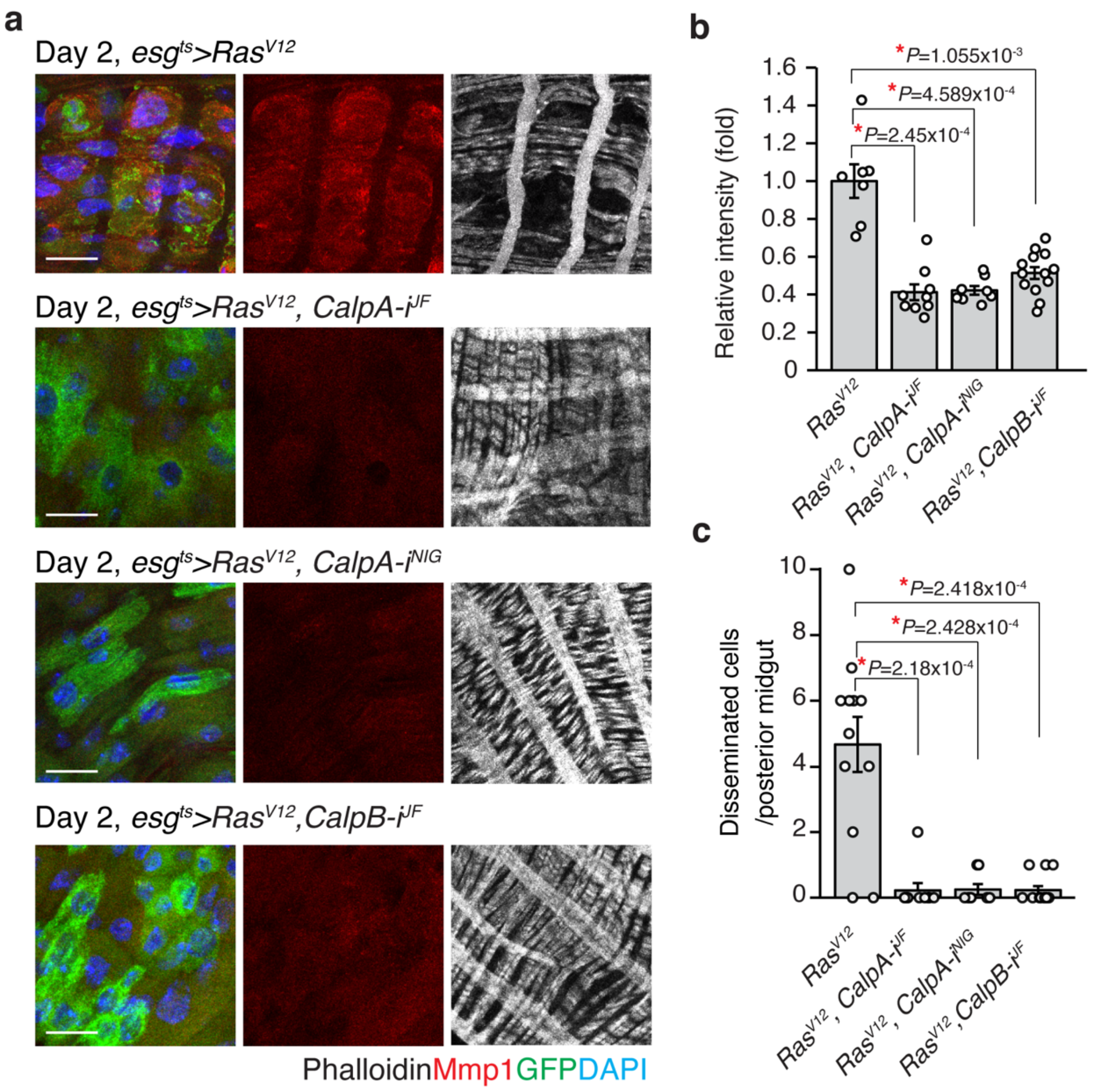

Supplementary Figure 10. *Calpain* knockdown in *Ras^{V12}* cells suppress Mmp1 expression and inhibits cell dissemination.

a, Representative images of Mmp1(red) expression levels and visceral muscle integrity after 2 days of transgene induction. Visceral muscle is stained with Phalloidin (gray). ISC/EB are marked with GFP (green), and nuclei are stained with DAPI (blue). Scale bar, 20μm. N=7 (*esg^{ts}>Ras^{V12}*), N=9 (*esg^{ts}>Ras^{V12}, CalpA-i^{JF}*), N=8(*esg^{ts}>Ras^{V12}, CalpA-i^{NIG}*), N=13 (*esg^{ts}>Ras^{V12}, CalpB-i^{JF}*) biological replicates. **b,** Quantification of Mmp1 levels per midgut. 3 regions were measured for each biological independent sample. **c,** Quantification of disseminated cells per midgut after 2 days of expression: N=12 (*esg^{ts}>Ras^{V12}*), N=9 (*esg^{ts}>Ras^{V12}, CalpA-i^{JF}*), N=8 (*esg^{ts}>Ras^{V12}, CalpA-i^{NIG}*), N=13 (*esg^{ts}>Ras^{V12}, CalpB-i^{JF}*) biological replicates. In **b** and **c**, mean±SEMs are shown with individual data points. Data were analyzed by two-tailed unpaired Student's *t*-test. Asterisks indicate statistical significance (*P<0.01), and P-values are indicated in graph.
