# Supplementary material for: Dissemination of RasV12-transformed cells requires the mechanosensitive channel Piezo
Source: Nat Commun. 2020 Jul 16;11:3568. doi: 10.1038/s41467-020-17341-y (PMC7366633; doi:10.1038/s41467-020-17341-y)
Supplement: Supplementary file 2 — Description of Additional Supplementary Files [file 41467_2020_17341_MOESM2_ESM.pdf]

Description of Additional Supplementary Files

File Name: Supplementary Movie 1

Description: Video of a representative *Ras*^{V12} cell generating blebs and ELEVs (z-projection of 25 µm stack, duration: 4 hours, 6 fps). Ras^{V12} was induced for 2 days with *esg*^{ts}. The cell is captured from Supplemental Video 3. Scale bar is 10 µm.

File Name: Supplementary Movie 2

Description: *Ex vivo* live imaging of day 2 *esg*^{ts} posterior midgut (z-projection of 25 µm stack, duration: 4 hours, 10 fps).

File Name: Supplementary Movie 3

Description: *Ex vivo* live imaging of day 2 *esg*^{ts}>*Ras*^{V12} posterior midgut (z-projection of 25 µm stack, duration: 4 hours, 5 fps). Arrowheads point to the sites of blebbing and ELEV formation.

File Name: Supplementary Movie 4

Description: *Ex vivo* live imaging of day 2 *esg*^{ts}>*Raf*^{gof} posterior midgut (z-projection of 25 µm stack, duration: 4 hours, 10 fps).

File Name: Supplementary Movie 5

Description: Bleb mediated dissemination of a *Ras*^{V12} cell in the posterior midgut (z-projection of 21 µm stack, duration: 2 hours 16 minutes, 10 fps). Arrowhead indicates blebbing. Arrow points out the detachment of the blebbing cell from the midgut epithelium.

File Name: Supplementary Movie 6

Description: Dissemination of *Ras*^{V12} cell in the posterior midgut (z-projection of 25.5 µm stack, duration: 1 hour 40 minutes, 5 fps). White arrow points to a cell that exits the midgut. Still shots are shown in Supplemental Figure 7.

File Name: Supplementary Movie 7

Description: *Ex vivo* live imaging of day 2 *esg*^{ts}>*Ras*^{V12}, *piezo-i*^{8486} posterior midgut (z-projection of 25 µm stack, duration: 4 hours, 10 fps).

File Name: Supplementary Movie 8

Description: *Ex vivo* live imaging of day 2 *esg*^{ts}>*Ras*^{V12}, *piezo-i*^{v2796} posterior midgut (z-projection of 25 µm stack, duration: 4 hours, 10 fps).

File Name: Supplementary Movie 9

Description: *Ex vivo* live imaging of day 2 *esg*^{ts}>*Ras*^{V12}, *piezo-i*^{8486}, *Cortactin-HA* posterior midgut (z-projection of 30 µm stack, duration: 4 hours, 10 fps).
