# Supplementary material for: Dissemination of RasV12-transformed cells requires the mechanosensitive channel Piezo
Source: Nat Commun. 2020 Jul 16;11:3568. doi: 10.1038/s41467-020-17341-y (PMC7366633; doi:10.1038/s41467-020-17341-y)
Supplement: Supplementary file 12 — Reporting Summary [file 41467_2020_17341_MOESM12_ESM.pdf]

nature research

Corresponding author(s): Young V. Kwon
Last updated by author(s): Jun 17, 2020

Reporting Summary

Nature Research wishes to improve the reproducibility of the work that we publish. This form provides structure for consistency and transparency in reporting. For further information on Nature Research policies, see Authors & Referees and the Editorial Policy Checklist.

Statistics

For all statistical analyses, confirm that the following items are present in the figure legend, table legend, main text, or Methods section.

n/a	Confirmed	
☐	☒	The exact sample size (*n*) for each experimental group/condition, given as a discrete number and unit of measurement
☐	☒	A statement on whether measurements were taken from distinct samples or whether the same sample was measured repeatedly
☐	☒	The statistical test(s) used AND whether they are one- or two-sided<br>*Only common tests should be described solely by name; describe more complex techniques in the Methods section.*
☒	☐	A description of all covariates tested
☒	☐	A description of any assumptions or corrections, such as tests of normality and adjustment for multiple comparisons
☐	☒	A full description of the statistical parameters including central tendency (e.g. means) or other basic estimates (e.g. regression coefficient) AND variation (e.g. standard deviation) or associated estimates of uncertainty (e.g. confidence intervals)
☒	☐	For null hypothesis testing, the test statistic (e.g. *F*, *t*, *r*) with confidence intervals, effect sizes, degrees of freedom and *P* value noted<br>*Give P values as exact values whenever suitable.*
☒	☐	For Bayesian analysis, information on the choice of priors and Markov chain Monte Carlo settings
☒	☐	For hierarchical and complex designs, identification of the appropriate level for tests and full reporting of outcomes
☒	☐	Estimates of effect sizes (e.g. Cohen's *d*, Pearson's *r*), indicating how they were calculated

Our web collection on statistics for biologists contains articles on many of the points above.

Software and code

Policy information about availability of computer code

Data collection	LAS X (Leica Microsystems) software for confocal imaging analysis and LIGHTNING (Leica Microsystems) software for high resolution image productions.
Data analysis	NIH Image J (Fiji version 1.52p), GraphPad Prism 8, 'R' software

For manuscripts utilizing custom algorithms or software that are central to the research but not yet described in published literature, software must be made available to editors/reviewers. We strongly encourage code deposition in a community repository (e.g. GitHub). See the Nature Research guidelines for submitting code & software for further information.

Data

Policy information about availability of data

All manuscripts must include a data availability statement. This statement should provide the following information, where applicable:

- Accession codes, unique identifiers, or web links for publicly available datasets
- A list of figures that have associated raw data
- A description of any restrictions on data availability

The authors declare that the data supporting the findings of this study are available within the paper and its supplementary information files.

Field-specific reporting

Please select the one below that is the best fit for your research. If you are not sure, read the appropriate sections before making your selection.

☒ Life sciences ☐ Behavioural & social sciences ☐ Ecological, evolutionary & environmental sciences

Life sciences study design

All studies must disclose on these points even when the disclosure is negative.

Sample size	Sample sizes were chosen empirically based on the observed effects and listed in the figure legends.
Data exclusions	No data was excluded.
Replication	All replications were successful.
Randomization	Randomization was not used.
Blinding	Blinding was not used.

Reporting for specific materials, systems and methods

We require information from authors about some types of materials, experimental systems and methods used in many studies. Here, indicate whether each material, system or method listed is relevant to your study. If you are not sure if a list item applies to your research, read the appropriate section before selecting a response.

Materials & experimental systems

n/a	Involved in the study	
☐	☒	Antibodies
☒	☐	Eukaryotic cell lines
☒	☐	Palaeontology
☐	☒	Animals and other organisms
☒	☐	Human research participants
☒	☐	Clinical data

Methods

n/a	Involved in the study	
☒	☐	ChIP-seq
☒	☐	Flow cytometry
☒	☐	MRI-based neuroimaging

Antibodies

Antibodies used

For immunohistochemistry, the primary antibodies used in this study were:
anti-GFP antibody, Alexa Fluor® 488 (1:1000; Thermo Fisher Scientific, A-21311; rabbit)
anti-Dl antibody (1:1000; Developmental Studies Hybridoma Bank, C594.9B; mouse)
anti-Mmp1 antibody (1:1000; Developmental Studies Hybridoma Bank, 3B8D12; mouse)
anti-phospho-histone H3 antibody (1:1000; Millipore, 06-570; rabbit; 1:1000; Abcam, ab14955; mouse)
anti-Laminin B1 antibody (1:1000; Abcam, ab47650; rabbit)
anti-HA antibody (1:2000; Santa Cruz, SC7392; mouse).

Secondary antibodies used in this study were:
anti-rabbit and anti-mouse IgGs conjugated to Alexa Flour® 594 or Alexa Fluor® 647 (1:1000; Thermo Fisher Scientific, A-11012, A11005, A-21244, A-21235; goat).

Filamentous Actin was stained with Phalloidin conjugated to Alexa Fluor® 594 or 647 (1:1000; Thermo Fisher Scientific, A-12381, A-22287).

Nuclei were stained with DAPI (1:2000; Sigma, D9542).

Validation

All antibodies used in this study have been tested by the company and have been cited by other authors.

Animals and other organisms

Policy information about studies involving animals; ARRIVE guidelines recommended for reporting animal research

Laboratory animals

Drosophila lines obtained from the Bloomington Drosophila Stock Center (BDSC) are the followings:
UAS-piezo-GFP/TM6B (#58773), UAS-RasV12 (III) (#4847), UAS-Rafgof (#2033), UAS-p35 (#5073), UAS-Lifeact-mRFP (#58362), UAS-Actin-mRFP (#24778), UAS-cortactin-HA (#9368), ey-GAL4 (#5534), and UAS-EGFP (#5430). We also used UAS-RasV12 (II) (laboratory stock). Cell dissemination phenotypes induced by our laboratory UAS-RasV12 (II) and BDSC UAS-RasV12 (III) (#4847) alleles were comparable. For the experiments to check subcellular distribution of Piezo-GFP and Cortactin-HA, we omitted UAS-GFP from esgts.
We used two piezo RNAi lines: 8486R-3 37 (shown as UAS-piezo-i8486) from the National Institute of Genetics, Japan (NIG-Fly; https://shigen.nig.ac.jp/fly/nigfly) and v2796 38 (shown as UAS-piezo-iv2796) from the Vienna Drosophila Resource Center (VDRC; www.vdrc.at).

	For other knockdown experiments, we obtained from BDSC: UAS-cortactin RNAi (BDSC #32871), UAS-Calpain-A RNAi (BDSC #29455), UAS-Calpain-B RNAi (BDSC #25963). We also used UAS-Calpain A RNAi (NIG #7563R-3) from NIG-Fly.
Wild animals	N/A
Field-collected samples	N/A
Ethics oversight	N/A

Note that full information on the approval of the study protocol must also be provided in the manuscript.
